# Gut Lactate Boosts *Ruminococcus* via Histone Lactylation to Mediate Time‐Restricted Feeding Protection in Crohn's Disease

**DOI:** 10.1002/advs.202518419

**Published:** 2026-04-07

**Authors:** Linwen Huang, Huishi Tan, Senhui Weng, Yuntao Liu, Lingxu Song, Shaoyu Cheng, Zelong Lin, Jiawei Chen, Fei Tan, Jun Wang, Jinke Huang, Linkun Cai, Jiwei Chai, Cailing Zhong, Yanqiang Shi, Wendi Zhang, Haiyan Zhang, Chongyang Huang

**Affiliations:** ^1^ Department of Gastroenterology The Second Affiliated Hospital of Guangzhou University of Chinese Medicine Guangzhou China; ^2^ Department of Gastroenterology and Hepatology Guangzhou Key Laboratory of Digestive Diseases Guangzhou Digestive Disease Center Guangzhou First People's Hospital School of Medicine South China University of Technology Guangzhou China; ^3^ Guangdong Provincial Key Laboratory of Chinese Medicine for Prevention and Treatment for Refractory Chronic Diseases State Key Laboratory of Dampness Syndrome of Chinese Medicine State Key Laboratory of Traditional Chinese Medicine Syndrome Guangzhou China; ^4^ Institute of Dermatology and Venereology Dermatology Hospital Southern Medical University Guangzhou China; ^5^ Department of Gastroenterology Guangdong Provincial Key Laboratory of Gastroenterology Institute of Gastroenterology of Guangdong Province Nanfang Hospital Southern Medical University Guangzhou China

**Keywords:** Crohn's disease, histone lactylation, lactate, *Ruminococcus*, time‐restricted feeding

## Abstract

**Background**: Crohn's disease (CD) is characterized by impaired epithelial barrier function and dysregulated gut microbiota, particularly characterized by a reduced abundance of short‐chain fatty acid (SCFA)‐producing bacteria such as *Ruminococcus*. Time‐restricted feeding (TRF), which limits daily food intake to specific feeding windows, has been demonstrated to restore microbial balance, enrich SCFA‐producing bacteria, and enhance gut homeostasis. Nevertheless, the extent to which TRF confers protection against CD, as well as the mechanisms underlying this effect, remains largely unexplored.

**Methods**: Fecal samples were obtained from patients with active CD and from healthy control subjects to quantify the abundance of *Ruminococcus*. Using 16S rRNA sequencing, we examined the impact of TRF regimens with varying fasting/feeding cycles (12/12, 16/8, and 20/4) on *Ruminococcus*. In preclinical CD models, we systematically evaluated the protective effect of TRF by analyzing colonic inflammation and fibrosis using histopathological techniques. RNA‐seq and epigenetic analyses were performed to elucidate the underlying mechanisms.

**Results**: Fecal *Ruminococcus* abundance was significantly reduced in patients with CD and inversely correlated with disease activity indices. Among the tested regimens, only the 12/12 fasting/feeding regimen (TRF_12h), but not regimens with shorter feeding windows, robustly increased the *Ruminococcus* abundance and potently stimulated mitochondrial β‐oxidation and lactate generation. Preemptive administration of TRF_12h markedly protected against colitis severity and fibrosis in CD models, as evidenced by marked reductions in F4/80^+^/MPO^+^ inflammatory cell infiltration and decreased extracellular matrix deposition. RNA‐seq analysis revealed that TRF‐mediated epithelial protection was predominantly driven by SCFA‐dependent activation of hypoxia‐inducible factor‐1α (HIF‐1α) signaling. IEC‐specific knockout of HIF‐1α largely abrogated the protective effects of TRF, confirming its essential role. Mechanistically, TRF‐induced gut‐derived lactate promoted histone H4K12 lactylation (H4K12la), leading to upregulation of SLC9A3 expression, which reinforced a localized acidic microenvironment conducive to *Ruminococcus* enrichment.

**Conclusions**: Collectively, TRF enriches SCFA‐producing *Ruminococcus* through a gut lactate‐driven histone lactylation‐SLC9A3 signaling axis, thereby alleviating inflammation and fibrosis in Crohn's disease under preventive conditions. *Rum*inococcus‐derived SCFA further enhances epithelial mitochondrial β‐oxidation and activates HIF‐1α‐dependent signaling pathways to strengthen the barrier integrity. This study provides a compelling mechanistic foundation for the clinical exploration of TRF as a therapeutic dietary strategy for patients with CD.

## Graphical abstract

TRF enriches *Ruminococcus*, a bacterial genus producing SCFAs, and activates the epithelial HIF‐1α signaling pathway. This mechanism protects the colonic mucosa from inflammatory insults in colitis models. Mechanistically, gut lactate production during starvation and refeeding mediates H4K12la, which increases SLC9A3 expression and creates an acidic gut microenvironment to support the rapid enrichment of *Ruminococcus*.

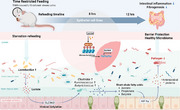

**Abbreviations**
ACOX1: Acyl‐CoA Oxidase 1
CD: Crohn's disease
CPT1/2: carnitine palmitoyltransferase 1/2
CXCL: C‐X‐C motif chemokine ligand
EHHADH: Enoyl‐CoA Hydratase And 3‐Hydroxyacyl CoA Dehydrogenase
GO: gene ontology
GSEA: gene set enrichment analysis
H&E: hematoxylin and eosin
HMGCS2: 3‐Hydroxy‐3‐Methylglutaryl‐CoA Synthase 2
IBD: Inflammatory Bowel Disease
IECs: Intestinal epithelial cells
IL: Interleukin
IOD: Integrated optical density
KEGG: Kyoto Encyclopedia of Genes and Genomes
LC‐MS: liquid chromatography‐mass spectrometry
LPS: lipopolysaccharide
MCAD: medium chain acyl‐CoA dehydrogenase
MMP: Matrix metalloproteinase
MUC2: mucin 2
NOS2: Nitric Oxide Synthase 2
PAS: Periodic Acid‐Schiff
PPI: protein–protein interaction
RNA‐seq: RNA sequencing
SLC27A2: Solute Carrier Family 27 Member 2
SLC5A8: Solute Carrier Family 5 Member 8
SLC9A3: Solute Carrier Family 9 Member 3
TNBS: 2,4,6‐Trinitrobenzenesulfonic acid
TNF‐α: Tumor Necrosis Factor α
ZT: Zeitgeber time

## 1 Background

Crohn's disease (CD) is a chronic, transmural inflammatory bowel disorder of the gastrointestinal tract characterized by dysbiosis, disruption of intestinal epithelial barrier integrity, and metabolic alterations [1, 2]. Dysregulated host‐microbiota interactions are central to immune imbalance, persistent inflammation, and progressive fibrogenesis, underscoring the urgent need for therapeutic strategies that target multiple, interconnected pathogenic pathways [3, 4, 5].

The gut microbiota play a fundamental role in maintaining intestinal homeostasis, and its disruption (dysbiosis) is strongly implicated in CD pathogenesis [6, 7]. Notably, patients with CD exhibited a marked depletion of short‐chain fatty acid (SCFA)‐producing *Clostridia*, including *Faecalibacterium prausnitzii* and *Ruminococcus bromii* [8, 9]. These commensals, particularly members of the *Ruminococcus* genus, are critical mediators of colonization resistance against enteric pathogens, largely through their capacity to produce SCFAs [10, 11]. SCFAs, including butyrate, acetate, and propionate, function as key metabolic and signaling molecules that enhance epithelial barrier function, suppress pro‐inflammatory signaling pathways, and maintain a physiologically hypoxic luminal microenvironment unfavorable for facultative anaerobes [12]. Accordingly, dietary strategies aimed at restoring SCFA‐producing microbial populations represent a promising and mechanistically grounded approach to CD management [13].

*Lactobacillus* species have been demonstrated to functionally synergize with SCFA‐producing bacteria, including *Clostridia*, thereby enhancing their abundance and functional efficacy [14, 15]. Experimental evidence suggests that *L. rhamnosus* and *L. murinus* can facilitate *Clostridiales* recovery after antibiotic‐induced microbiota disruption, promoting robust butyrate production and nutrient competition that suppresses pathogens [16]. Lactate, a major metabolic end product of *Lactobacillus* fermentation, has recently emerged as more than an energy source. Notably, lactate serves as a critical precursor for histone lactylation, a newly characterized epigenetic modification that directly influences gene expression [17, 18]. Despite these insights, the functional significance of lactate‐mediated cross‐talk between *Lactobacillus* and SCFA‐producing bacteria such as *Ruminococcus* remains largely unexplored.

Dietary interventions, including exclusive enteral nutrition or the Mediterranean diet, have gained substantial clinical interest as non‐invasive approaches to modulate gut microbiota and alleviate inflammation in CD [19, 20]. Time‐restricted feeding (TRF), which restricts daily food intake to specific windows, has been demonstrated to exert beneficial effects on systemic metabolism and gut microbial composition [21, 22, 23]. Emerging evidence further suggests that TRF can enrich SCFA‐producing microbiota and improve disease outcomes in neurodegenerative conditions such as Alzheimer's disease [24]. However, whether TRF modulates *Ruminococcus* abundance and confers protective effects in patients with CD remains unclear.

In this study, we identify a previously unrecognized mechanism by which TRF prevents and mitigates CD‐like intestinal inflammation through the orchestration of a novel lactate‐H4K12la‐SLC9A3 signaling axis that selectively promotes *Ruminococcus* enrichment and activates epithelial HIF‐1α signaling. We demonstrate that a specific 12/12 h fasting/feeding cycle uniquely optimizes this pathway, thereby providing a robust mechanistic rationale for TRF as a targeted dietary strategy in CD management. Importantly, the identification of the lactate‐H4K12la‐SLC9A3 signaling axis as a key determinant of *Ruminococcus* enrichment represents a groundbreaking advance, addressing a critical gap in our understanding of how TRF exerts its protective effects in CD.

## 2 Methods

### 2.1 Human Sample Collection

Fecal samples were collected from patients with active Crohn's disease (n = 30) and from healthy controls (n = 20) recruited through the Department of Gastroenterology. All patients with CD were either newly diagnosed or experiencing a disease relapse and had no pharmacological treatment, including antibiotics, corticosteroids, immunomodulators, or biologics, for ≥ 4 weeks before sample collection. The cohort included patients with inflammatory (B1, n = 18), stricturing (B2, n = 9), and penetrating (B3, n = 3) disease phenotypes, according to the Montreal classification. This study protocol complied with the ethical principles outlined in the Declaration of Helsinki, and written informed consent was obtained from all participants. Participant characteristics are summarized in Table S5. To minimize the dietary confounding effects of gut microbiota composition, all participants, including both patients with CD and healthy controls, completed a 7‐day food frequency questionnaire. Individuals reporting substantial changes in habitual diet, consumption of probiotics/prebiotics, or adherence to specialized dietary regimens (including exclusive enteral nutrition, or a low FODMAP diet) within the preceding 3 months were excluded. This exclusion period was selected to minimize the confounding effects of recent dietary modifications on the established gut microbiota communities. Clinical disease activity was evaluated using a standardized, multimodal assessment, incorporating clinical symptoms, endoscopic observations, histopathological analysis, and radiological criteria. The Clinical Research Ethics Committee of the Second Affiliated Hospital of Guangzhou University of Chinese Medicine granted ethical approval (No. BF2023‐008‐01).

### 2.2 Animal Models

Wild‐type C57BL/6J mice were obtained from the Animal Experiment Center of Guangzhou University of Chinese Medicine. IL‐10 knockout (IL‐10‐KO) mice were obtained from GemPharmatech Co., Ltd. (Jiangsu), and *Hif* *1a* ^flox/flox^ *Vil1*‐Cre mice were obtained from Cyagen Biosciences. All mice were housed under specific‐pathogen‐free conditions within the barrier facility of the Animal Experiment Center at Guangzhou University of Chinese Medicine. Animal procedures were conducted in strict compliance with animal welfare ethical principles and were approved by the Animal Welfare and Ethics Review Committee of the Animal Experiment Center, Guangzhou University of Chinese Medicine (Approval No. 20241022003).

IEC‐specific HIF‐1α knockout mice (*Hif1a* ^ΔIEC^) were generated by crossing *Hif1a* ^flox/flox^ mice (C57BL/6J‐*Hif1a* ^*em* *1* *Cf* *l* *ox*^/Cya, Cyagen #CKOCMP‐15251) with Vil1‐Cre mice to assess HIF‐1α function in intestinal epithelial cells (IECs). Intestinal epithelial‐specific deletion was confirmed by PCR (Table S6). PCR products were separated by agarose gel electrophoresis. Western blot demonstrated significant HIF‐1α reduction in intestinal tissues with β‐actin as a loading control.

### 2.3 Time‐Restricted Feeding (TRF) Regimens

To examine the effect of feeding‐fasting cycles on gut microbiota and colitis, wild‐type C57BL/6J mice were subjected to one of three distinct TRF regimens for a period of ≥ 4 weeks before and throughout subsequent experimental procedures. Mice were assigned to four experimental groups: TRF_12h group: 12‐h feeding window followed by a 12‐h fast (from Zeitgeber time (ZT) 2 to ZT 14). TRF_16h group: 8‐h feeding window followed by a 16‐h fast (from ZT 2 to ZT 18). TRF_20h group: 4‐h feeding window followed by a 20‐h fast (from ZT 2 to ZT 22); and a normal diet (ND) control group with* ad libitum* access to food. These specific fasting/feeding windows were selected based on established protocols in metabolic and circadian biology studies [21, 22, 25], and represent a graded spectrum of dietary restriction, ranging from moderate (12/12 h) to severe (20/4 h). The 16/8 h regimen, which is commonly applied in human TRF studies, was included for translational relevance [22]. The 12 h window was selected as a baseline physiological threshold, as it aligns with the natural light/dark cycle and is sufficient to trigger metabolic switch following hepatic glycogen depletion [26, 27]. The 16 and 20 h windows were included to assess whether extended fasting would confer dose‐dependent benefits or impose excessive physiological stress [28]. After four weeks of dietary intervention, we evaluated the effects of each regimen on microbial composition and host metabolism.

### 2.4 Fasting‐Refeeding

To investigate the dynamic effects of fasting and refeeding on HIF‐1α signaling and downstream metabolic pathways, 6–8‐week‐old wild‐type C57BL/6J mice were randomly divided into four groups: (1) Stay Hungry: 12‐h fasting with immediate sampling; (2) Refeed 4h: 12‐h fasting followed by 4‐h refeeding; (3) Refeed 8h: 12‐h fasting followed by 8‐h refeeding; (4) Refeed 12h: 12‐h fasting followed by 12‐h refeeding. Colon tissues were harvested for HIF‐1α and downstream gene expression analysis by qPCR, and cecal contents were obtained for gut microbiota profiling.

### 2.5 The Chronic Colitis Model

The chronic 2,4,6‐Trinitrobenzenesulfonic acid (TNBS)‐induced colitis model was established according to a previously published protocol [29]. This chemical TNBS model was selected due to its well‐characterized ability to recapitulate the interplay between chronic inflammation and *de novo* intestinal fibrosis, a hallmark complication of CD, and its high reproducibility and controllable nature in inducing transmural, granulomatous inflammation [29, 30]. Briefly, TNBS sensitization solution was prepared by mixing acetone/olive oil (4:1, v/v) with 5% (w/v) TNBS at a 4:1 ratio (v/v), yielding 2.5% TNBS. For colitis induction, the working solution was prepared by combining equal volumes of 5% (w/v) TNBS and anhydrous ethanol. Male C57BL/6J mice (6–8 weeks old) were epidermally sensitized with 100 µL sensitization solution to the shaved dorsal skin. Seven days post‐sensitization, chronic inflammation was induced by intrarectal administration of 80 µL TNBS‐ethanol solution under isoflurane anesthesia, followed by weekly administrations for six consecutive weeks. Body weight and survival were monitored daily. Before colitis induction, mice underwent a 4‐week time‐restricted feeding (TRF) regimen and were assigned to ND (ad libitum), TRF‐12 (12‐h feeding/12‐h fasting), TRF‐16 (8‐h feeding/16‐h fasting), or TRF‐20 (4‐h feeding/20‐h fasting) groups. TRF was maintained throughout the 6‐week modeling period, resulting in a total experimental duration of 10 weeks.

Furthermore, the IL‐10 knockout (IL‐10^−/−^) model was utilized to validate our findings in a spontaneous, immune‐mediated model of chronic colitis that more closely mimics the chronic inflammatory and microbial features of human CD [31]. Notably, 3‐month‐old male littermate IL‐10‐KO mice were randomly assigned to two groups: Normal diet (ND) with ad libitum feeding, and TRF‐12h with 12‐h feeding/12‐h fasting daily. By 5 months of age, IL‐10‐KO mice developed typical colitis manifestations, including weight loss, diarrhea, and rectal prolapse. After 3 months of dietary intervention, serum and tissue samples were collected at 6 months of age for analysis.

In these chronic colitis models, repeated inflammatory insults lead to significant morbidity and mortality, reflecting severe transmural or systemic inflammation [29, 30]. Herein, survival rates were employed to evaluate overall colitis severity in this experimental setting.

For the analysis of fibrosis‐associated protein expression, tissue sampling from the distal colon was performed using a standardized protocol to ensure inter‐sample consistency [32]. Following the chronic TNBS regimen, the entire colon was excised, gently flushed with cold PBS, and opened longitudinally. The primary fibrotic stricture was identified based on macroscopic features, including palpable thickening, rigidity, and/or luminal stenosis. From the core region of the contiguous fibrotic lesion, three adjacent full‐thickness tissue strips were collected, pooled, and immediately flash‐frozen in liquid nitrogen as a single composite sample, then stored at −80°C until analysis.

### 2.6 TNBS‐Induced Acute Colitis Models

The acute TNBS‐induced colitis model was established following a previously published protocol [29], with detailed reagent information provided in Table S7. Mice were sensitized identically to the protocol as previously described, and seven days later, acute colitis was induced by a single intrarectal administration of 100 µL of 2.5% (w/v) TNBS in 50% ethanol under isoflurane anesthesia. Mice were monitored for 72 h, with daily assessments of disease progression. Colon tissues were subsequently collected for length measurement and histological scoring.

To evaluate the effects of *Ruminococcus bromii* (RB) and lactate on TNBS‐induced acute colitis, frozen RB stocks were resuscitated and cultured anaerobically at 37°C for 16–24 h. Bacterial suspensions with OD600 = 0.5–0.6 (log phase, 1 × 10^9^ CFU/mL) were aliquoted into 1 × 10^9^ CFU/mouse/day in 200 µL PBS for gavage. Control and TNBS‐only mice received sterile PBS. The 6–8‐week‐old C57BL/6J mice were randomly divided into: (1) Control (PBS i.g., daily), (2) lactate (sodium lactate 300 mg/kg i.g., daily), (3) *R.bromii* (*R.bromii* i.g., daily), (4) TNBS (PBS i.g., daily), (5) TNBS+lactate (sodium lactate 300 mg/kg i.g., daily), (6) TNBS+*R.bromii* (*R.bromii* i.g., daily), (7) TNBS+*R.bromii*+lactate (*R.bromii*+sodium lactate 300 mg/kg i.g., daily). Agents were administered daily starting 3 days pre‐TNBS induction until tissue collection. Colon tissues were harvested 72 h after TNBS administration for analysis.

### 2.7 Microbiota and Metabolomics

We applied 16S rRNA gene sequencing to quantify the gut microbiome composition of fecal samples, with sequencing services provided by Metabo‐Profile Biotechnology (Shanghai, China). In brief, we collected mouse fecal samples from ND and TRF‐12h groups prior to TNBS model establishment, and total genomic DNA was extracted using the OMEGA Soil DNA Kit (M5635‐02). Genomic DNA (2 µL) was amplified via PCR with primers targeting the V3–V4 region: 338F (5′‐ACTCCTACGGGAGGCAGCA‐3′) and 806R (5′‐GGACTACHVGGGTWTCTAAT‐3′). After purification, sequencing libraries were constructed using the Illumina TruSeq Nano DNA LT Library Prep Kit. Libraries passing quality control were sequenced on the Illumina NovaSeq platform with the NovaSeq 6000 SP Reagent Kit (500 cycles). The sequencing data were filtered, and high‐quality sequences were denoised, merged, and chimeras were removed to generate Amplicon Sequence Variants (ASVs) and an ASV table. Based on the ASV results and taxonomic annotation, we performed analyses of alpha diversity (Chao1, Shannon), beta diversity (UniFrac distance), and LEfSe for intergroup differences using QIIME2 and R software. Statistical significance for beta diversity was assessed using PERMANOVA with 999 permutations, and multiple testing correction for differential abundance analysis was performed using the Benjamini‐Hochberg false discovery rate (FDR) method.

We employed UPLC‐MS/MS for metabolomic analysis of targeted short‐chain fatty acids in the feces of mice that underwent four weeks of TRF intervention. Sample preparation, metabolite extraction, and LC‐MS/MS detection were performed at Metabo‐Profile Biotechnology (Shanghai) Co., Ltd. Briefly, fecal samples (5 mg) were homogenized by adding 20 µL of water, extracted with 120 µL methanol containing an internal standard followed by centrifugation, and then 40 µL of derivatization reagent was added (30°C, 60 min). After a dilution‐centrifugation process, the supernatant was collected and mixed with the internal standard for analysis. UPLC‐MS/MS analyses were performed on an ACQUITY UPLC‐Xevo TQ‐S system (Waters) using an ACQUITY UPLC BEH C18 column (1.7 µm analytical column with VanGuard pre‐column). A multistep gradient elution (0–12 min, 12%–100% B) was applied using mobile phase A (0.1% formic acid in water) and B (acetonitrile) in ESI‐ mode (2.0 kV, 550°C). Samples were analyzed randomly, and mixed quality control samples were inserted every 14 samples to ensure analytical reliability. Peak integration and quantification were performed using MassLynx software (v4.1), while statistical analyses, including PCA, OPLS‐DA, and univariate tests, were conducted using iMAP software (v1.0), developed by Metabo‐Profile.

### 2.8 Histology and Immunostaining

Colonic samples from mice were fixed in 4% paraformaldehyde for 24 h, paraffin‐embedded, and sectioned at 5 µm. Sections were stained with hematoxylin‐eosin (H&E), periodic acid‐Schiff (PAS), Masson's trichrome, or Sirius Red following manufacturer's protocols (Servicebio, China). Colitis severity in H&E‐stained sections was assessed in a blinded manner by two independent pathologists using a well‐established semi‐quantitative scoring system [29, 30]. The detailed scoring system is listed in Tables S8 and S9. The scores for each parameter were summed to generate an average histopathology score for each sample. The percentage of fibrotic area was quantified using ImageJ software. Goblet cell counts were determined by counting PAS‐positive goblet cells per crypt, with the average calculated from five crypts in the mouse colon.

For immunohistochemistry (IHC) and immunofluorescence (IF), deparaffinized sections underwent endogenous peroxidase quenching, antigen retrieval, and blocking. Sections were incubated overnight at 4°C with primary antibodies (see Table S10 for complete antibody information, including catalog numbers and dilutions) followed by a 2‐h incubation at room temperature with species‐specific biotinylated secondary antibodies (for IHC) or fluorophore‐conjugated secondary antibodies (for IF). Images were obtained using an inverted Nikon ECLIPSE Ti2‐E microscope, and protein expression was quantified using Image‐Pro Plus software (version 6.0; Media Cybernetics).

### 2.9 RNA Sequencing

Total RNA was isolated from mouse colon tissues using Trizol reagent. RNA integrity was assessed through three methods: (1) agarose gel electrophoresis (28S/18S rRNA ratio≈2:1), (2) Nanodrop measurement (A260/A280 = 1.8–2.0; A260/A230 = 2.2), and (3) microcapillary electrophoresis (Agilent 2100 Bioanalyzer, RIN≥7). Transcriptomic libraries were prepared using the Hieff NGS Ultima kit (Yeasen, 12309ES): poly(A)+ RNA was enriched with Oligo(dT) magnetic beads and fragmented; double‐stranded cDNA was synthesized via reverse transcription; DNA ends were repaired, adenylated, and ligated to Illumina adapters; libraries were bead‐purified and PCR‐amplified. Qualified libraries underwent 150‐bp paired‐end sequencing on the Illumina NovaSeq X Plus system (Gene Denovo Biotechnology Co., Ltd., Guangzhou, China). Bioinformatic analysis was performed using the Omicsmart platform (http://www.omicsmart.com). Differential gene expression analysis was performed using the DESeq2 package in R. Genes with an adjusted *p*‐value (FDR)< 0.05 and an absolute fold change>1.5 were considered differentially expressed. Functional enrichment analysis, including GO (Gene Ontology) and KEGG (Kyoto Encyclopedia of Genes and Genomes) pathway analysis, was carried out to understand the biological functions and pathways associated with the differentially expressed genes. Heatmaps were generated to visualize the expression patterns of the differentially expressed genes. The normalized count data of significantly differentially expressed genes were transformed by row‐wise Z‐score normalization to clearly depict relative expression changes across experimental groups.

### 2.10 RT‐qPCR

Quantitative reverse transcription PCR (RT‐qPCR) was performed to evaluate transcriptional changes in inflammatory mediators, fibrotic markers, and metabolic regulators. Total RNA was extracted from snap‐frozen colon tissues and cells using TRIzol Reagent (AGBIO, Hunan, China; AG21101), followed by cDNA synthesis with RT‐gDNA Digestion Mix (Yeasen, Shanghai, China; 11151ES60). PCR amplification was performed using Hieff UNICON qPCR SYBR Green Master Mix (Yeasen, Cat# 11185ES08) on a LightCycler 96 System (Roche). Gene expression levels were calculated using the 2^^−ΔΔCt^ method, 18S rRNA was used as the reference gene for normalization. Detailed primer sequences are provided in Table S11.

### 2.11 Immunoblotting

Colon tissues and cells were lysed in RIPA buffer (Beyotime, P0013G) and protein concentrations were determined by BCA assay (Thermo Scientific, 23225). Samples were separated by 7.5%–15% gradient SDS‐PAGE and transferred to PVDF membranes (Millipore). After blocking with 5% non‐fat milk for 1–2 h, membranes were incubated with primary antibodies at 4°C overnight, followed by HRP‐conjugated secondary antibodies for 1 h at room temperature. Protein bands were detected using ECL substrate (Yeasen, 36222ES76) and quantified with ImageJ software, normalized to β‐actin/GAPDH. Antibodies are detailed in Table S10.

### 2.12 CUT&Tag Library Construction

The CUT&Tag assay was performed using the Hyperactive CUT&Tag Kit for Illumina (Vazyme, TD903‐TD904) according to the manufacturer's instructions at OE Biotech Co., Ltd. (Shanghai, China). Concanavalin A‐coated magnetic beads (ConA beads) bound cells, followed by permeabilization with non‐ionic digitonin. Bead‐bound cells were sequentially incubated with primary antibody (Anti‐L‐Lactyl‐Histone H4 (Lys12), PTM Biolabs), secondary antibody, and Hyperactive pA‐Tn5 Transposase to cleave protein‐bound DNA; Tn5 transposase simultaneously fragmented DNA and ligated P5/P7 adapters. Libraries were amplified via PCR using P5/P7 primers, purified, quality‐assessed on an Agilent 2100 Bioanalyzer, and sequenced as 150‐bp paired‐end reads on an Illumina NovaSeq 6000. Data analysis comprised: adapter trimming/quality filtering with fastp to generate clean reads; Bowtie2 alignment to the reference genome; SEACR peak calling (stringent mode) identifying protein‐binding regions; ChIPseeker annotation of peak‐associated genes/genomic features; differential binding analysis using MAnorm, which performed regression modeling of M‐value (log_2_[TRF‐12h/ND]) and A‐value (log_2_[TRF‐12h×ND]) based on common peaks, normalized systemic bias in M/A‐values, and computed p‐values. Statistically significant differential peaks satisfied *p*‐value < 0.05 and |M‐value| > 1. Visualization utilized *R* and IGV v2.19.5.

### 2.13 Multiplex Bead‐Based Immunoassay of Serum Inflammatory Cytokines

Peripheral blood from IL‐10^−^/^−^ mice was collected in serum separator tubes, and serum was isolated by centrifugation. Serum levels of inflammatory factors, including IL‐1β, TNF‐α, CXCL1, and CCL2 were quantified using a multiplex bead‐based immunoassay. According to the instructions for the XMplex Mouse multiPlex Custom Panel Kit (Item No. XMplex03240771), 5 µL of antibody‐conjugated microspheres were added per well, followed by 50 µL of standards or serum samples. After incubation at 37°C for 1 h, magnetic washing was performed. Subsequently, 50 µL of detection antibody was added and incubated at 37°C for 0.5 h, followed by a wash step. Then, 50 µL of phycoerythrin (PE)‐conjugated detection reagent was added and incubated at 37°C for 15 min in the dark. Finally, 55 µL of wash buffer was added, and median fluorescence intensity (MFI) was measured with the XM‐BIOTECH XMplex‐100 instrument (ABclonal Technology, Wuhan, China). Cytokine concentrations were calculated based on standard curves.

### 2.14 Bacterial Strain and Culture Conditions

*Ruminococcus bromii* strain ATCC 27255 (Cat. No. B225615; Moore et al.) was purchased from Ningbo Mingzhou Biotechnology Co., Ltd. This strain was selected as it can degrade resistant starch and produce butyrate and is a well‐characterized type strain. It represents the SCFA‐producing *Ruminococcus* genus, which was depleted in CD patients. Cryopreserved stocks in 20% (v/v) glycerol were maintained at −80°C. For revival, stocks were inoculated into CMC broth supplemented with 15% (v/v) rumen fluid, 5 µg/mL hemin, and 50 µg/mL vitamin K_1_. The base medium was autoclaved at 121°C for 30 min, cooled to <50°C, and supplements were added aseptically. Cultures were grown in an anaerobic atmosphere of 85% N_2_, 5% H_2_, and 10% CO_2_ at 37°C for 24–48 h.

### 2.15 DNA Extraction and Bacterial Quantification

Bacterial DNA was extracted from human stool samples using the QIAamp Fast DNA Stool Mini Kit (51604, Qiagen) and from mouse stool samples using the MolPure Stool DNA Kit (18820ES, YEASEN), both according to the manufacturers' protocols. Bacterial quantification was performed by quantitative real‐time PCR (qPCR) on an ABI QuantStudio 7 Flex using the 2X SYBR Green Pro Taq HS Premix (AG11735). The universal bacterial 16S rRNA gene was amplified as an endogenous reference to normalize the quantification of specific bacterial targets in stool samples. Detailed primer sequences are provided in Table S12.

### 2.16 Cell Culture

IEC‐6 cells were cultured in Dulbecco's Modified Eagle's Medium (DMEM; Gibco) supplemented with 10% fetal bovine serum (Pricella), 100 U/mL penicillin, and 100 µg/mL streptomycin at 37°C under 5% CO_2_. Intestinal epithelial IEC‐6 cells were treated with D‐lactate or L‐lactate (20 mm). The detailed information of reagents is listed in Table S7.

### 2.17 Statistical Analysis

All statistical analyses were performed using GraphPad Prism software (Version 9.0). Data are presented as mean ± standard deviation (SD) or mean ± standard error of the mean (SEM). Comparisons between two groups were conducted using a two‐tailed Student's t‐test, whereas comparisons among multiple groups were analyzed using a one‐way analysis of variance (ANOVA). Statistical significance was defined as a *p*‐value < 0.05. For high‐dimensional omics datasets, false discovery rate (FDR) correction was applied to adjust for multiple testing.

Additional methodological details are provided in the Supporting Information.

## 3 Results

### 3.1 Reduced Fecal *Ruminococcus* Abundance Correlates With Disease Activity in Patients With CD

**FIGURE 1:**
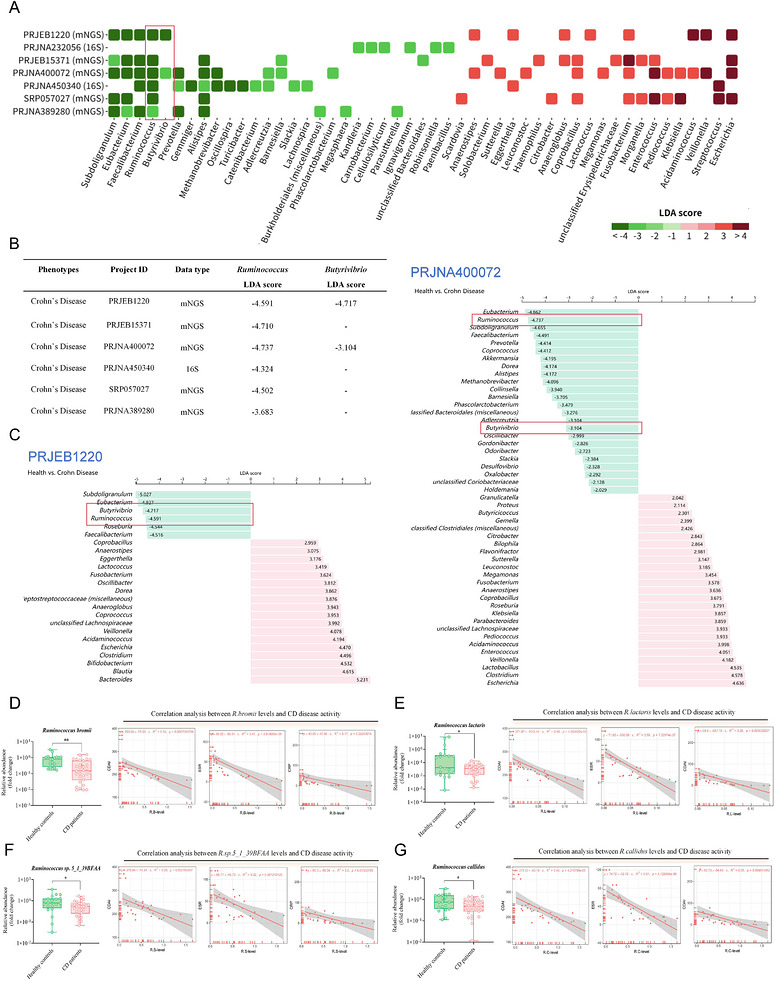
Decreased *Ruminococcus* abundance in the stool samples of patients with CD. (A) Relative abundance of *Ruminococcus* and *Butyrivibrio* genera in the stool samples of patients with CD, analyzed using available 16S or mNGS data from the GMrepo database. (B) Linear discriminant analysis (LDA) score (Log_10_) of *Ruminococcus* and *Butyrivibrio* genus in different cohorts. (C) LDA score histogram demonstrating *Ruminococcus* and *Butyrivibrio* abundance in cohorts (PRJEB1220; PRJNA400072). (D–G) Quantitative PCR detection of *Ruminococcus* species (*Ruminococcus bromii*, *Ruminococcus callidus*, *Ruminococcus lactaris*, and *Ruminococcus sp. 5_1_39BFAA*) in the feces of 30 patients with active CD and 20 healthy controls. Correlations between *Ruminococcus* levels and CDAI scores, as well as serum C‐reactive protein (CRP) and erythrocyte sedimentation rate (ESR) in patients with CD, were assessed using Pearson correlation analyses. Data: Graphs represent mean ± standard error of the mean (SEM). For comparisons between two groups, a two‐tailed unpaired Student's *t*‐test was used. For comparisons among more than two groups, one‐way analysis of variance (ANOVA) was performed. Statistical significance was defined as *p < 0.05, **p < 0.01. The sample size (n) for each experiment is indicated in the figure legends and represents biological replicates.

We first assessed the abundance of *Ruminococcus* in patients with CD and its correlation with disease activity. Metagenomic analysis of fecal samples from GMrepo cohorts revealed a significant reduction in *Ruminococcus* abundance in 6 of 7 CD cohorts (with Project IDs: PRJEB1220, PRJEB15371, PRJNA400072, PRJNA450340, SRP057027, and PRJNA389280). In comparison, the abundance of the *Butyrivibrio* genus was reduced in two cohorts (PRJEB1220 and PRJNA400072) (Figure 1A–C). We further analyzed differential species within the *Ruminococcus* genus that contribute to this reduction. Specifically, the SCFA‐producing species *Ruminococcus bromii* (*R.bromii*) was consistently depleted across cohorts, along with other species such as *Ruminococcus callidus* (*R.callidus*), *Ruminococcus lactaris* (*R.lactaris*), *R* *uminococcus sp. 5_1_39BFAA*, and other unclassified *Ruminococcus spp*. (Tables S1–S4). These findings underscore the critical role of *Ruminococcus*, particularly *R.bromii*, in maintaining gut homeostasis and its potential depletion in CD pathogenesis.

Consistent with these findings, qPCR analysis of fecal samples from patients with active CD (n = 30) and healthy controls (n = 20) confirmed significantly reduced *Ruminococcus* levels in CD (Figure 1D–G). This reduction was observed across all CD subtypes (B1, B2, and B3) (Figure S1A) and correlated negatively with disease activity scores (CDAI), serum CRP, and ESR levels (Figure 1D–G). Notably, individuals with severe CD (CDAI > 200) demonstrated markedly reduced levels of *Ruminococcus*, underscoring its inverse association with disease severity. Collectively, these results suggest that dietary strategies aimed at enriching *Ruminococcus* may represent a promising therapeutic avenue for CD management.

### 3.2 TRF Enriches *Ruminococcus* and Enhances Host Mitochondrial β‐Oxidation

**FIGURE 2:**
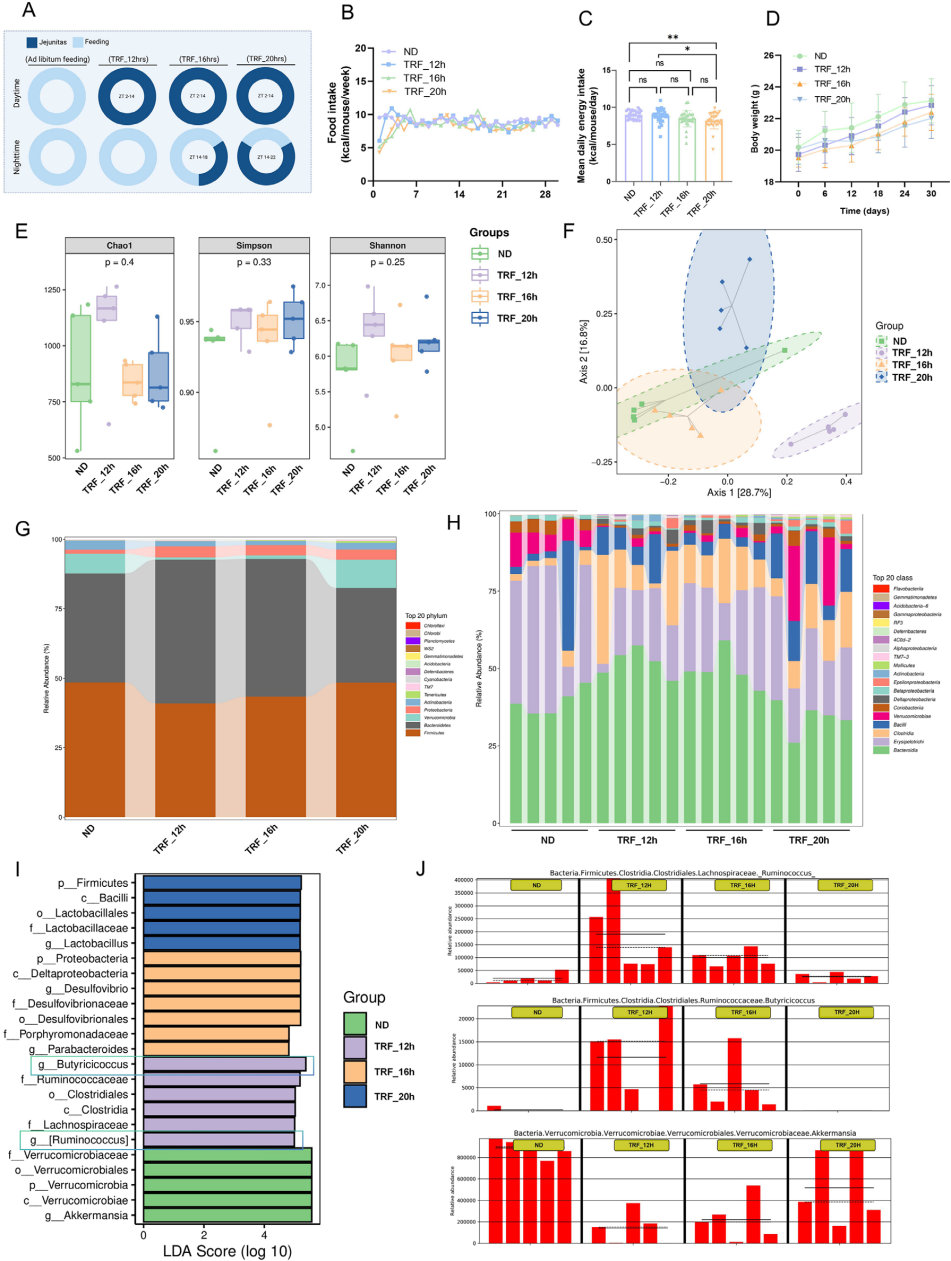
TRF enhances microbiota diversity and enriches the *Ruminococcus* genus. (A) Experimental design of TRF regimens (Mice fed a normal diet ad libitum or TRF diets with 12/12, 16/8, and 20/4 fasting/feeding cycles were designated as ND, TRF_12h, TRF_16h, and TRF_20h, respectively). (B) Food intake was recorded every day (n = 6 mice per group). (C) Mean daily energy intake was recorded every day (n = 6 mice per group). (D) Body weight changes were recorded every 6 days. All experimental groups demonstrated comparable body weights. (E) α‐Diversity indices (Chao1, Simpson, and Shannon) of fecal microbiota (n = 5 mice per group). (F) PCoA plot (Bray‐Curtis distance) demonstrating microbiota clustering (n = 5 mice). (G) Phylum‐level taxonomic composition (Presented as group; Top 3 altered phyla: *Bacteroidetes*, *Firmicutes*, and *Verrucomicrobia*). (H) Class‐level taxonomic composition (Presented as sample; Top 3 altered classes: *Bacteroidia*, *Erysipelotrichi*, and *Clostridia*). (I) LEfSe analysis identifying enriched genera (LDA score > 3.0). (J) Differentially enriched genera and their tendency to change between groups are presented as a histogram plot. Data: Graphs represent mean ± standard deviation (SD). 16S rRNA Microbiota Data: α‐diversity (Chao1, Shannon, and Simpson indices): Compared between groups using Kruskal–Wallis test with Dunn's post‐hoc test. Differential Abundance Analysis (LEfSe): LDA scores were calculated, and the threshold for significance was set at an LDA score > 3.0. The non‐parametric factorial Kruskal–Wallis sum‐rank test was used to identify features with significant abundance differences, followed by the Wilcoxon rank‐sum test for pairwise comparisons. The resulting *p*‐values from LEfSe were adjusted using the Benjamini–Hochberg (BH) method.

Fasting and feeding cycles are known to affect gut microbiota composition and enrich SCFA‐producing bacteria [24, 33]. To systematically evaluate the impact of fasting duration on gut microbiota and host metabolism, we initially designed three representative TRF regimens (12/12 h, 16/8 h, and 20/4 h) based on established circadian and metabolic research protocols [21, 25]. To determine whether TRF modulates *Ruminococcus* abundance, C57BL/6J mice were subjected to 4‐week TRF regimens with different fasting/feeding cycles (12/12, 16/8, 20/4) or a normal diet (ND) (Figure 2A). Energy intake was comparable among ND, TRF_12h (12/12 fasting/feeding cycles), and TRF_16h (16/8 fasting/feeding cycles) groups, but reduced in TRF_20h (20/4 fasting/feeding cycles) mice (Figure 2B,C). Body weight remained stable across all groups before TNBS administration (Figure 2D).

Fecal microbial composition was then analyzed by 16S rRNA gene sequencing. Before dietary intervention, no significant differences in gut microbiota composition were observed among groups (Figure S2A,B). However, TRF_12h significantly altered gut microbiota composition, increasing α‐diversity as measured by Chao1, Simpson, and Shannon indices (Figure 2E; Figure S2C). Principal coordinates analysis (PCoA) based on Bray‐Curtis distance revealed a distinct clustering of the TRF_12h‐fed group compared with other dietary regimens (Figure 2F). At the phylum level, TRF_12h‐fed mice exhibited a higher relative abundance of *Bacteroidetes* and a lower relative abundance of *Verrucomicrobia* compared with ND‐fed mice (Figure 2G; Figure S2D). LEfSe analysis identified *Ruminococcus* and *Butyricicoccus* as significantly enriched genera in the TRF_12h mice (LDA score > 3.0 and BH‐adjusted *p* < 0.05) (Figure 2H–J). Moreover, KEGG and MetaCyc pathway analysis demonstrated that TRF_12h enhances microbial lipid metabolism and fatty acid biosynthesis (Figure S2E).

**FIGURE 3:**
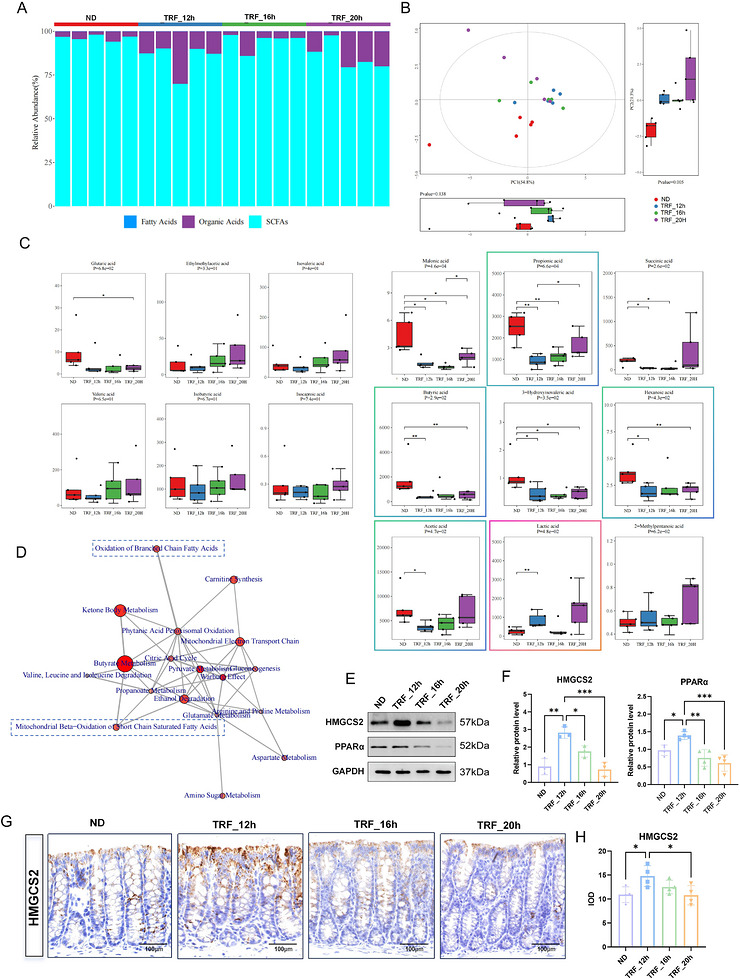
TRF enhances mitochondrial β‐oxidation of SCFA in intestinal epithelium. (A) Relative abundance of fatty acids, organic acids, and SCFAs was presented as a histogram plot. (B) PCoA plot demonstrating fecal metabolites clustering (n = 5 mice). (C) Fecal metabolite levels, including butyrate, acetate, propionate, and lactate, via LC‐MS (n = 5 mice). (D) Pathway enrichment of differential fecal metabolites. (E, F) Immunoblotting analysis of PPARα and HMGCS2 protein expression and quantitative results (n = 3 mice). (G) Immunohistochemical staining of HMGCS2 in colonic epithelium (brown: HMGCS2; scale bar: 20 µm). (H) Quantitative analysis of HMGCS2‐positive stained areas. Data: Graphs represent mean ± SD. Comparisons were made by two‐tailed *t*‐test and one‐way ANOVA; *p < 0.05, **p < 0.01 versus the ND group. Data were combined from three independent experiments.

Targeted metabolomics was performed to quantify fecal metabolites after 4 weeks of TRF. TRF_12h and TRF_20h significantly altered SCFA‐related metabolic profiles, with higher overall organic acid levels compared with ND‐fed mice (Figure 3A). PCoA revealed a clear separation of metabolic profiles between TRF groups and ND controls (Figure 3B). Unexpectedly, metabolomic analysis demonstrated reduced fecal concentrations of SCFAs (butyrate, acetate, and propionate) but elevated lactate levels in TRF_12h and TRF_20h groups (Figure 3C). Concurrently, elevated fecal lactate in the TRF_20h group coincided with pronounced expansion of *Lactobacillus* (Figure 3C). TRF_12h‐fed mice also exhibited a moderate lactate increase, suggesting a duration‐dependent relationship between feeding windows and lactate production (Figure S3A).

The decline in luminal SCFAs likely reflects enhanced epithelial utilization by the host. Consistently, metabolomic pathway analysis indicated that TRF augmented mitochondrial β‐oxidation of fatty acids and oxidation of branched‐chain fatty acids (Figure 3D; Figure S3B,C). β‐oxidation (*Cpt1/2, Mcad, Acox1, Ehhadh, Slc27a2, and Hmgcs2*) and ketogenesis (*Fgf21 and Pparα*) genes were significantly upregulated in the colonic epithelium of TRF_12h‐fed mice compared to ND controls (Figure S3D) [34, 35]. Immunoblotting and IHC confirmed the increased protein expression of HMGCS2 and PPARα in the colonic epithelium (Figure 3E–H), indicating that TRF enhances the host epithelial oxidation of SCFAs.

### 3.3 TRF Attenuates Colitis Severity and Fibrosis in Murine CD Models

**FIGURE 4:**
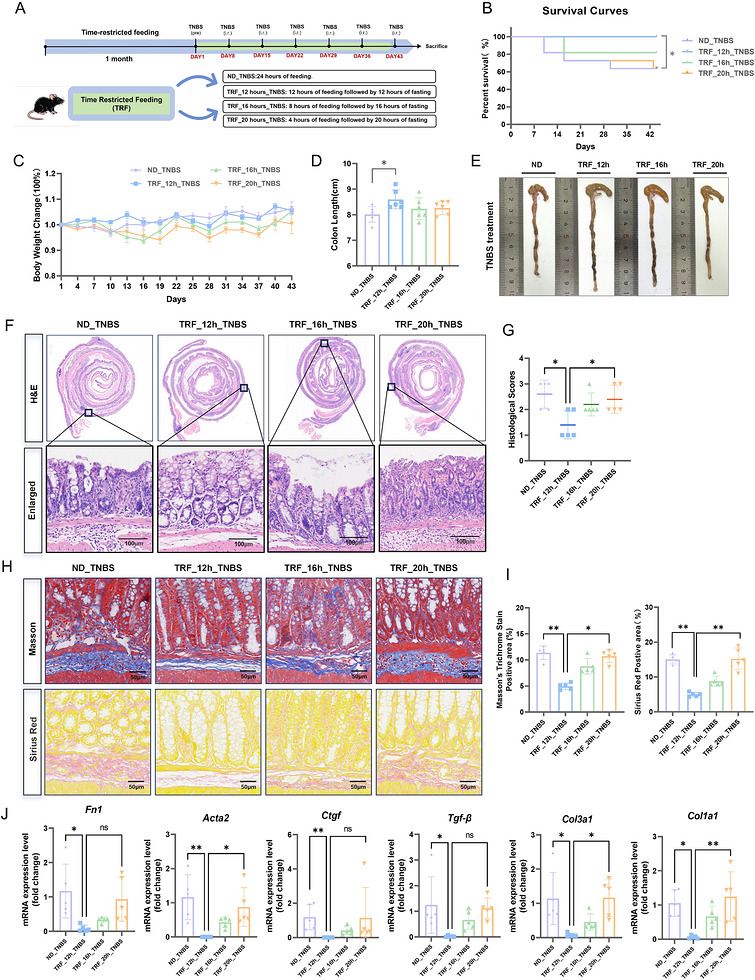
TRF attenuates TNBS‐induced colitis severity and intestinal fibrosis. (A) Mice exposed to TRF regimens with indicated fasting/feeding cycles and a normal diet were then subjected to TNBS‐induced chronic colitis. (B) Survival curves of mice subjected to different TRF regimens post‐TNBS challenge (n = 10 mice per group). (C) Body weight changes were monitored throughout the experimental period. (D, E) Colon length quantification (n = 6 mice per group). (F) Representative H&E staining of colonic tissues (scale bar: 100 µm). (G) Histopathological scoring of epithelial injury and immune infiltration (n = 5 mice per group). (H) Representative image of Masson's trichrome and Sirius red staining (blue/red: collagen deposition; scale bar: 50 µm). (I) Quantification of collagen‐positive areas (n = 5 mice per group). (J) Relative mRNA expression of several pro‐fibrotic genes (*Fn1, ACTA2, CTGF, TGF‐β, COL3A1*, and *COL1A1*) was measured using qRT‐PCR (n = 5 mice per group). Note: The survival curves presented reflect the severity of the TNBS‐induced chronic colitis model, in which repeated inflammatory challenges lead to significant morbidity and mortality. Data: Graphs represent mean ± SD. Comparisons were made by two‐tailed *t*‐test and one‐way ANOVA; *p < 0.05, **p < 0.01. ns, not significant. Survival curves were analyzed using the log‐rank test. Data were combined from three independent experiments.

To evaluate the protective effects of TRF, mice fed with TRF regimens were subjected to TNBS‐induced CD‐like colitis (Figure 4A). All experimental groups demonstrated comparable baseline body weights before TNBS administration. However, only TRF_12h improved survival and attenuated colon shortening and histopathological injury (Figure 4B–G). This protection was accompanied by a downregulation of pro‐inflammatory genes (*Tnf*, *Il‐1b*, *Il‐6*, and *Nos2*) and chemokines (*Ccl4*, *Ccl20*, and *Cxcl10*) (Figure S4A), as well as reduced immune cell infiltration, including F4/80^+^ monocytes and MPO^+^ neutrophils (Figure S4B–E). Importantly, TRF_12h enhanced intestinal barrier integrity, as evidenced by increased Muc2/PAS^+^ goblet cells (Figure S5A,B) and upregulation of tight junction proteins (Occludin, ZO‐1) (Figure S5C,D). These findings indicate that TRF_12h suppresses inflammation and strengthens the intestinal barrier, conferring protection against TNBS‐induced colitis.

**FIGURE 5:**
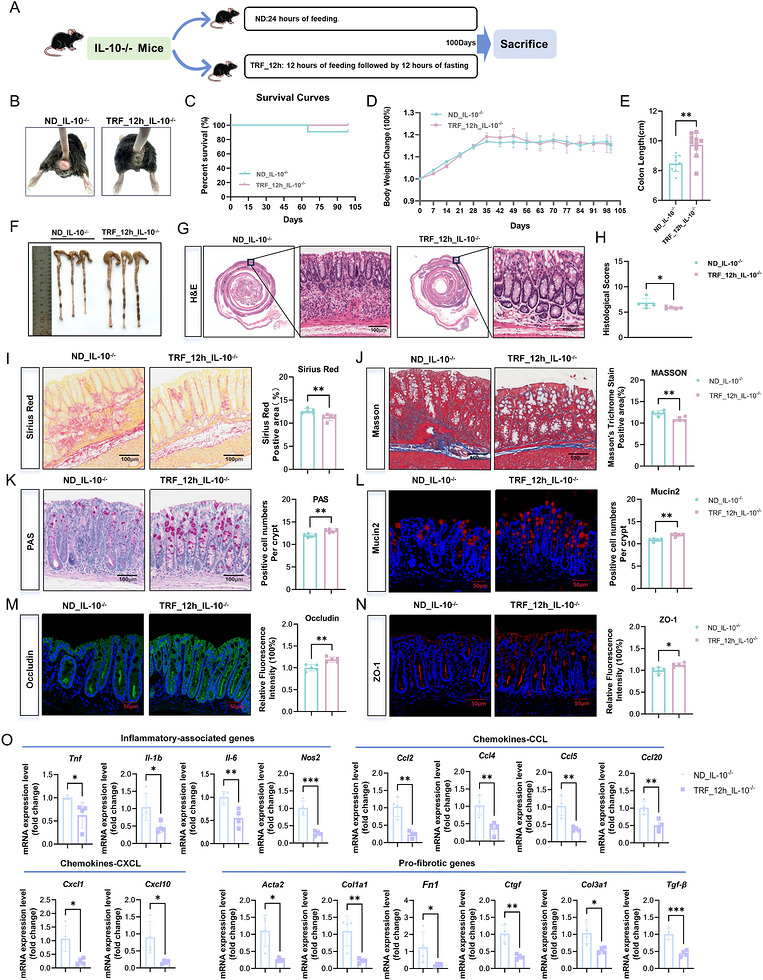
TRF protects from IL‐10 deficiency‐induced colitis and fibrogenesis. (A) Experimental design of TRF regimens (IL‐10^−/−^ mice received a normal diet and TRF diets with 12/12 fasting/feeding cycles). (B) Symptoms of rectal prolapse in IL‐10^−/−^ mice. (C) Survival curves of mice subjected to different TRF regimens (n = 10 mice per group). (D) Body weight changes were monitored weekly. (E, F) Mice were sacrificed, and colon lengths were quantified (n = 9–10 mice per group). (G) Representative H&E staining of colonic tissues (scale bar: 100 µm). (H) Histopathological scoring of epithelial injury and immune infiltration (n = 5 mice per group). (I) Representative image of Sirius red staining (red: collagen deposition; scale bar: 50 µm) and quantification of collagen‐positive areas (n = 5 mice per group). (J) Representative image of Masson's trichrome (blue: collagen deposition; scale bar: 50 µm) and quantification of collagen‐positive areas (n = 5 mice per group). (K) Representative image of PAS staining (purple; scale bar: 50 µm) and quantification of PAS‐positive cell numbers (n = 5 mice per group). (L) Representative image of MUC2 staining (red; scale bar: 50 µm) and quantification of MUC2‐positive cell numbers (n = 5 mice per group). (M) Representative image of occludin staining (green; scale bar: 50 µm) and quantification of occludin‐positive areas (n = 5 mice per group). (N) Representative image of ZO‐1 staining (red; scale bar: 50 µm) and quantification of ZO‐1‐positive areas (n = 5 mice per group). (O) Relative mRNA expression of pro‐inflammatory (*TNF, IL‐1β, IL‐6, NOS2, CCL2*, *CXCL10*, and others) and pro‐fibrotic genes (*Fn1, ACTA2, CTGF, TGF‐β, COL3A1*, and *COL1A1*) was measured using qRT‐PCR (n = 4 mice per group). Data: Graphs represent mean ± SD. Comparisons were made by two‐tailed *t*‐test and one‐way ANOVA; *p < 0.05, **p < 0.01 versus the ND group. Survival curves were analyzed using the log‐rank test. Data were combined from three independent experiments.

TRF_12h also attenuated intestinal fibrosis, a hallmark of CD, as demonstrated by decreased collagen deposition (Masson's trichrome/Sirius red) and downregulation of fibrogenic genes, including collagen type I alpha 1 (*Col1a1*), collagen type III alpha (*Col3a1*), actin alpha 2 (*Acta2*), and fibronectin (Figure 4H–J). TRF_12h consistently downregulated profibrogenic growth factors such as *Tgf‐β* and *Ctgf* in the TNBS‐treated colon (Figure 4J). Comparable protection was observed in female mice, as evidenced by improved survival, reduced weight loss, and less colon shortening (Figure S6A–F). In addition, TRF_12h alleviated colonic histopathological damage and downregulated pro‐inflammatory (*Tnf, Il‐1b, Il‐6*, and *Nos2*) and pro‐fibrotic (*Col1a1, Col3a1, Acta2*, and *Fn1*) gene expression in female mice (Figure S6G–I). Consistent with the microbial and metabolic profiling data, TRF_12h uniquely improved survival rates and markedly attenuated colonic inflammation and fibrosis. Thus, the 12 h TRF regimen was selected for all subsequent mechanistic studies based on both microbial enrichment and in vivo phenotypic outcomes. Here, TRF_12h also significantly reduced colitis severity, improving survival, reducing colon shortening, and alleviating histopathological injury in IL‐10^−/−^ mice, a well‐established animal model resembling human IBD (Figure 5A–H). Notably, qRT‐PCR demonstrated suppressed expression of pro‐inflammatory and pro‐fibrotic genes (Figure 5O). Serum inflammatory cytokines, including IFN‐γ, IL‐1β, CXCL1, CCL5, and IL‐6, were significantly reduced in the TRF_12h group (Figure S7A). Histological staining confirmed reduced collagen deposition and restored barrier integrity (Figure 5I–N).

To determine whether TRF_12h‐induced gut microbiota changes were causally associated with colitis protection, we performed a fecal microbiota transplantation (FMT) in antibiotic‐pretreated mice, using donor microbiota from either TRF_12h‐fed or ND‐fed groups via oral gavage (Figure S8A). TRF_12h‐FMT recipients developed significantly milder colitis than ND‐FMT recipients, demonstrating reduced weight loss, longer colon length, and lower histopathological scores (Figure S8B–F). This protection was associated with enhanced expression of the barrier markers, including Mucin 2 and Occludin (Figure S8G,H), and downregulation of pro‐inflammatory and pro‐fibrotic genes in the colon (Figure S8I). These results directly demonstrate that TRF_12h‐shaped gut microbiota mediate protection against colitis.

### 3.4 TRF Activates Epithelial HIF‐1α Signaling to Protect Against TNBS‐Induced Colitis

We then performed single‐cell RNA sequencing (scRNA‐seq) to investigate the mechanism underlying TRF‐mediated protection against colitis. Unsupervised clustering analysis identified 20 distinct cell clusters in the colon, which were annotated using canonical marker genes (Figure S9A,B). These clusters included epithelial cells (Cluster 2/4/8 marked by Vil1, MUC, and KRT), immune cells such as macrophages (Cluster 3, marked by Lyz2/CD68), and neutrophils (Cluster 1, marked by CXCL2, CXCR2, and S100A8/9) (Figure S9C–E). Importantly, TRF_12h increased the proportion of epithelial cells (Clusters 2 and 8) and decreased immune cell clusters, particularly macrophages (Cluster 3) and neutrophils, in TNBS‐treated colons (Figure S9F). KEGG enrichment analysis of neutrophils (Cluster 1) and macrophages revealed that TRF_12h significantly downregulated pathways related to the TNF signaling pathway and Chemokine signaling pathway, which are critical for pro‐inflammatory responses (Figure S10A,B). Besides, differential gene expression analysis within epithelial cells revealed that TRF_12h significantly upregulated genes involved in fatty acid metabolism signaling as well as tight junction (Figure S10C,D). These results demonstrate that TRF_12h enhanced colonic barrier integrity and reduced immune cell infiltration and activation, which might collectively contribute to the mitigation of colitis.

**FIGURE 6:**
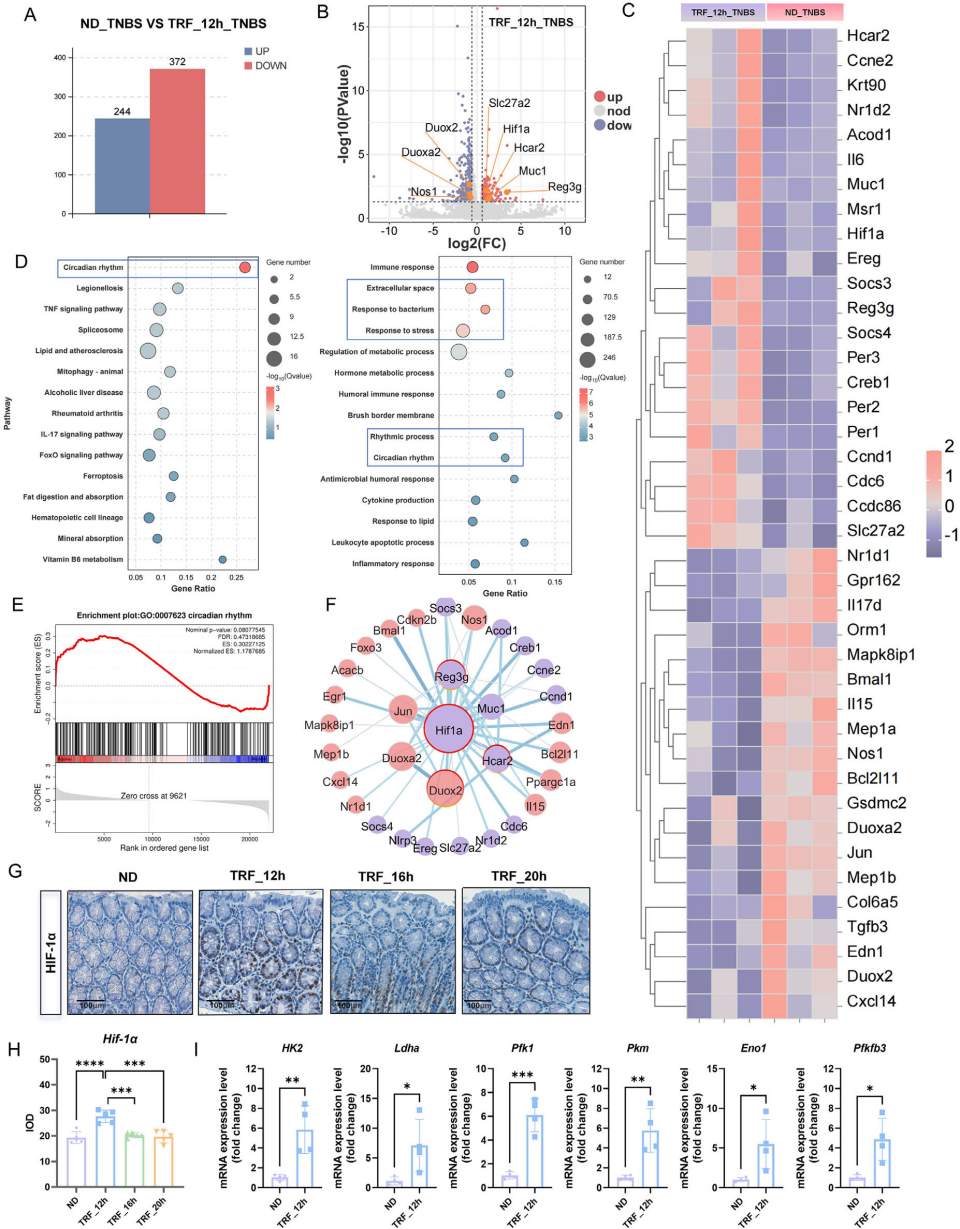
TRF mediates intestinal barrier protection by activating epithelial HIF‐1α signaling. (A) Overall differential gene expression numbers were displayed as a histogram. (B) Volcano plot demonstrating the significantly changed genes in the TNBS‐treated colon between TRF_12h‐fed and normal diet groups. (C) Heatmap representation of differentially expressed genes (circadian‐related gene expression profiles) in colonic tissues of mice subjected to ND and TRF regimens. Each row represents a gene, and the expression values are presented as row‐wise Z‐scores to standardize the data and highlight inter‐group differences. The color scale indicates relative expression levels (blue: below mean; red: above mean). (D) KEGG and GO enrichment pathway analyses in TRF‐treated mice compared to the normal diet group. (E) GSEA snapshot of GO pathway enrichment analysis: Circadian rhythms. (F) PPI network of differential expressed genes. (G) IHC analysis of HIF‐1α‐positive cells in colonic tissues (n = 5 mice). (H) Quantitative analysis of HIF‐1α‐positive stained areas. (I) qRT‐PCR analysis of HIF‐1α‐regulated genes, including *HK2, LDHA, PFK‐1, PKM, PFKFB3*, and *ENO1* (n = 4 per group). Data: Graphs represent mean ± SD. Comparisons were made by two‐tailed *t*‐test and one‐way ANOVA; *p < 0.05, **p < 0.01, ***p < 0.001, ****p < 0.0001. Data were combined from three independent experiments.

RNA sequencing revealed 616 differentially expressed genes (FDR < 0.05 and |log2FC| > 1) in TRF_12h‐fed mice post‐TNBS (244 upregulated, 372 downregulated) (Figure 6A,B). Kyoto encyclopedia of genes and genomes (KEGG)/gene ontology (GO) pathway analysis highlighted significant enrichment of circadian rhythm‐related pathways, with TRF upregulating core clock genes (*CREB1* and *PER1/2/3*) while suppressing *BMAL1* and *NR1D1* (Figure 6C–E). This temporal reprogramming was accompanied by enhanced expression of barrier‐associated genes (*Muc1*, *Reg3g*, *Ccnd1*, and *Ccne2*) and reduced expression of oxidative stress‐related genes (*Duox2*, *Duoxa2*, and *Nos1*) as well as pro‐apoptotic genes (*Bcl2l11* and *Jun*) (Figure 6C). Notably, we observed a significant upregulation of HIF‐1α expression specifically within the colonic tissues of TRF_12h‐fed mice following TNBS administration compared with ad libitum‐fed controls. Furthermore, RNA‐seq analysis indicated an upregulation of HCAR2, a key SCFA receptor mediating host‐specific responses, in the colonic tissues of TRF_12h‐fed mice (Figure 6C). REG3G, an antimicrobial peptide secreted by IECs, exhibits circadian rhythmicity and plays a critical role in shaping gut microbiota composition [36]. Conversely, DUOX2/DUOXA2, which catalyze the production of reactive oxygen species (ROS), were downregulated, potentially preserving anaerobic commensal bacteria [37]. Consistent with our RNA‐seq findings, Immunohistochemical (IHC) staining confirmed that TRF_12h treatment significantly reduced the epithelial expression of CCL20 and DUOX2, while upregulating HCAR2 and REG3G specifically within the intestinal epithelium (Figure S11A–D). These data reinforce that IECs are primary responders to TRF, coordinating a transcriptional program that dampens inflammation, enhances microbial sensing, and strengthens barrier function. RNA‐seq further demonstrated that TRF_12h upregulated epithelial cell‐proliferation markers, including CCND1, CCNE2, and CDC6. Consistently, IHC staining for PCNA and Ki67 showed that TRF enhances epithelial regeneration compared with control groups following TNBS challenge (Figure S11E,F). This demonstrates that TRF not only protects the epithelium but also actively enhances its regenerative capacity after inflammatory injury.

Protein–protein interaction (PPI) analysis identified a regulatory network linking transcriptional factor HIF‐1α to enhanced barrier effectors (HCAR2, MUC1, REG3G) and suppressed ROS‐producing enzymes (DUOX2/DUOXA2), positioning epithelial HIF‐1α signaling activation as a central mediator of TRF efficacy (Figure 6F). HIF‐1α is a well‐established regulator of intestinal epithelial barrier function and its stability and activity are known to be modulated by microbial metabolites, particularly SCFAs. Given that our earlier data showed TRF augmented mitochondrial β‐oxidation of SCFAs and upregulated the SCFA receptor HCAR2, we reasoned that HIF‐1α might serve as a key downstream effector linking TRF‐induced metabolic changes to epithelial protection. Indeed, IHC analysis confirmed that TRF_12h treatment significantly increased the number of HIF‐1α‐positive cells in the intestinal epithelium (Figure 6G,H). TRF_12h also upregulated canonical HIF‐1α target genes, including *lactate dehydrogenase A* (*LDHA*) and *pyruvate dehydrogenase kinase 1*, in TRF_12h‐fed mice (Figure 6I). Co‐staining with lineage‐specific markers, including sPLA2, LGR5, FABP1, and CHGA, revealed that HIF‐1α expression was predominantly localized to FABP1‐positive enterocytes, sPLA2‐positive Paneth cells and LGR5‐positive intestinal stem cells (Figure S12A).

### 3.5 Epithelial HIF‐1α Ablation Abolishes TRF Protection and Exacerbates Colitis

To directly assess the requirement of epithelial HIF‐1α, we generated mice with IEC‐specific HIF‐1α knockout (HIF‐1α^ΔIEC^) (Figure S13A–D). Following TRF or standard feeding, mice were subjected to TNBS‐induced colitis. IEC‐specific deletion of HIF‐1α completely abolished TRF‐mediated protection, leading to persistent weight loss, shorter colon length, and higher histopathological scores, thereby demonstrating the indispensable role of epithelial HIF‐1α signaling (Figure S14A–E). We further examined whether *Ruminococcus* and its metabolic SCFAs confer protection against intestinal inflammation. Indeed, butyrate administration activated epithelial HCAR2/HIF‐1α signaling and attenuated colitis severity in wild‐type but not HIF‐1α^ΔIEC^ mice (Figure S15A–F). This was evidenced by similar weight loss tendency, colon shortening, and histopathological scores compared to vehicle‐treated HIF‐1α^ΔIEC^ mice following TNBS challenge (Figure S15A–E). Furthermore, butyrate did not downregulate DUOX2/DUOXA2 or CCL20 expression within the colonic tissues of HIF‐1α^ΔIEC^ mice (Figure S15G).

**FIGURE 7:**
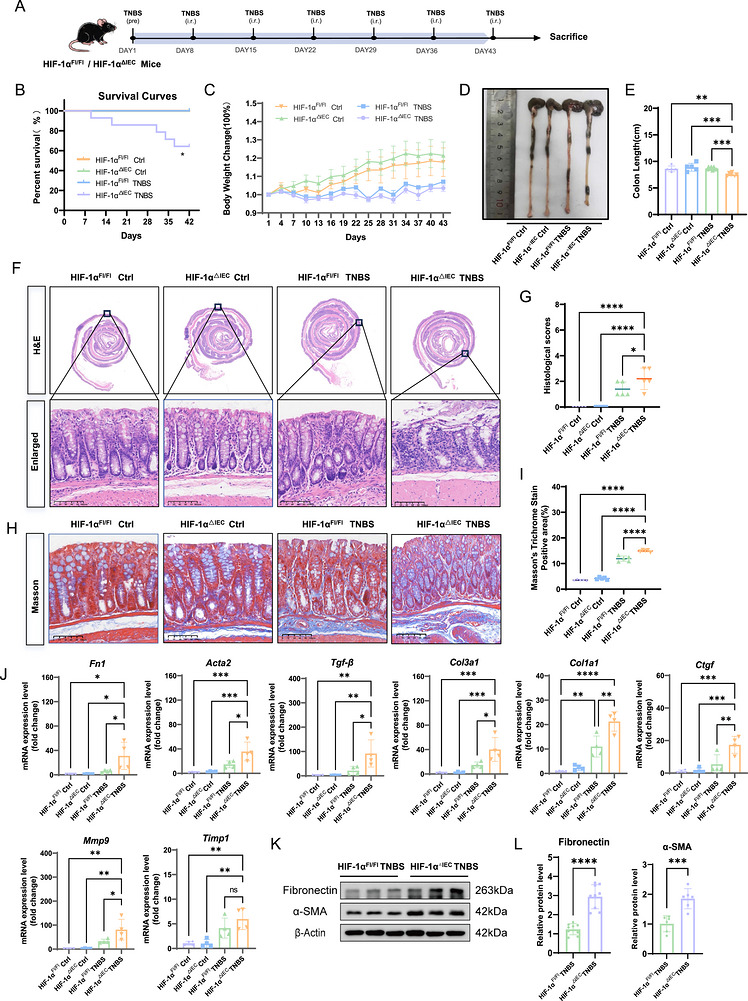
IEC‐specific HIF‐1α ablation exacerbates TNBS‐induced colitis and fibrosis. (A) Experimental design of HIF‐1α^ΔIEC^ versus WT littermates subjected to TNBS‐induced colitis modeling. (B) Survival curves of HIF‐1α^ΔIEC^ versus WT littermates post‐TNBS (n = 10 mice per group). (C) Body weight changes were monitored throughout the experimental period. (D, E) Colon length quantification (n = 5 mice). (F, G) Representative images of H&E‐stained colonic tissues (scale bar: 100 µm) and pathological scores (n = 5 mice per group). (H, I) Representative image of Masson's trichrome and Sirius red staining (blue/red indicates collagen deposition; scale bar: 50 µm), along with the quantification of collagen‐positive areas (n = 5 mice per group). (J) Relative mRNA expression of several pro‐fibrotic genes (*Fn1, ACTA2, CTGF, TGF‐β, COL3A1*, and *COL1A1*) was measured using qRT‐PCR (n = 4 mice per group). (K, L) Immunoblotting analysis of α‐SMA and Fibronectin protein expression and quantitative results (n = 3 mice). Data: Graphs represent mean ± SEM. Comparisons were made by two‐tailed *t*‐test and one‐way ANOVA; *p < 0.05, **p < 0.01, ***p < 0.001, ****p < 0.0001. Survival curves were analyzed using the log‐rank test. Data were combined from three independent experiments.

To further investigate the mechanism underlying HIF‐1α‐mediated barrier improvement, HIF‐1α^ΔIEC^ mice and their wild‐type littermates underwent TNBS‐induced chronic colitis (Figure 7A). HIF‐1α^ΔIEC^ mice demonstrated more severe intestinal inflammation than wild‐type controls after TNBS challenge, as indicated by survival rate, body weight loss, and colon length (Figure 7B–E). Histological analysis demonstrated that HIF‐1α^ΔIEC^ mice had increased inflammatory cell infiltration and crypt loss in colon tissues after TNBS treatment (Figure 7F,G). HIF‐1α^ΔIEC^ mice exhibited more severe intestinal fibrogenesis, evidenced by greater Masson's trichrome staining for collagen in TNBS‐treated colons (Figure 7H,I). Consistent with these findings, elevated expression of fibrogenic genes, including *Col1a1*, *Col3a1*, *Mmp9* and *Timp1*, were observed in the colonic tissues of HIF‐1α^ΔIEC^ mice following TNBS treatment (Figure 7J). Immunoblotting confirmed increased colonic fibronectin and α‐SMA expression (Figure 7K,L). Notably, qRT‐PCR results confirmed upregulation of pro‐inflammatory cytokine genes (*Tnf, Il‐1b, and Il‐6*) in HIF‐1α^ΔIEC^ mice post‐TNBS (Figure S16A).

Colonic tissues were harvested for RNA‐seq to identify differential gene expression (Figure S17A–C). As expected, HIF‐1α^ΔIEC^ mice demonstrated reduced glycolysis (ENO1, PFKFB3, and ALDOC). RNA‐seq also revealed disrupted barrier function with downregulated colonic N‐Cadherin, α‐defensin, and BCL2 Interacting Protein 3 in HIF‐1α^ΔIEC^ mice following TNBS treatment (Figure S17B). GO pathway analysis revealed that the immune system process and several immune response signaling pathways were involved (Figure S17D). In particular, pathways related to CCR6 chemokine receptor binding, regulation of monocyte chemotaxis, and keratinization were enriched in HIF‐1α^ΔIEC^ mice compared with WT mice (Figure S17E). PPI analysis revealed key proteins and interactions in pathways, with chemokine CCL20 expression increased in HIF‐1α^ΔIEC^ colon post‐TNBS treatment (Figure S17F). Epithelial HIF‐1α deletion exacerbates colitis and fibrogenesis by downregulating several signaling pathways, including glycolysis, tight junctions, defensin generation, and autophagy (Figure S17G). These findings support the role of *Ruminococcus* enrichment and the HIF‐1α signaling activation in mediating TRF's protection in TNBS‐induced chronic colitis.

### 3.6 Gut Lactate Promotes *Ruminococcus* Enrichment During Fasting‐Refeeding

We further investigated the mechanistic association between TRF and *Ruminococcus* enrichment, focusing on lactate‐mediated ecological niche remodeling. Previous studies have revealed that starvation‐refeeding increases *Lactobacillus* abundance and lactic acid production [38]. Consistently, metabolomic analysis revealed increased fecal lactate levels in TRF_20h‐fed mice (4‐h refeeding period), although this increase did not reach statistical significance. In contrast, TRF_12h (12‐h refeeding period) resulted in significant fecal lactate accumulation accompanied by robust *Ruminococcus* enrichment.

**FIGURE 8:**
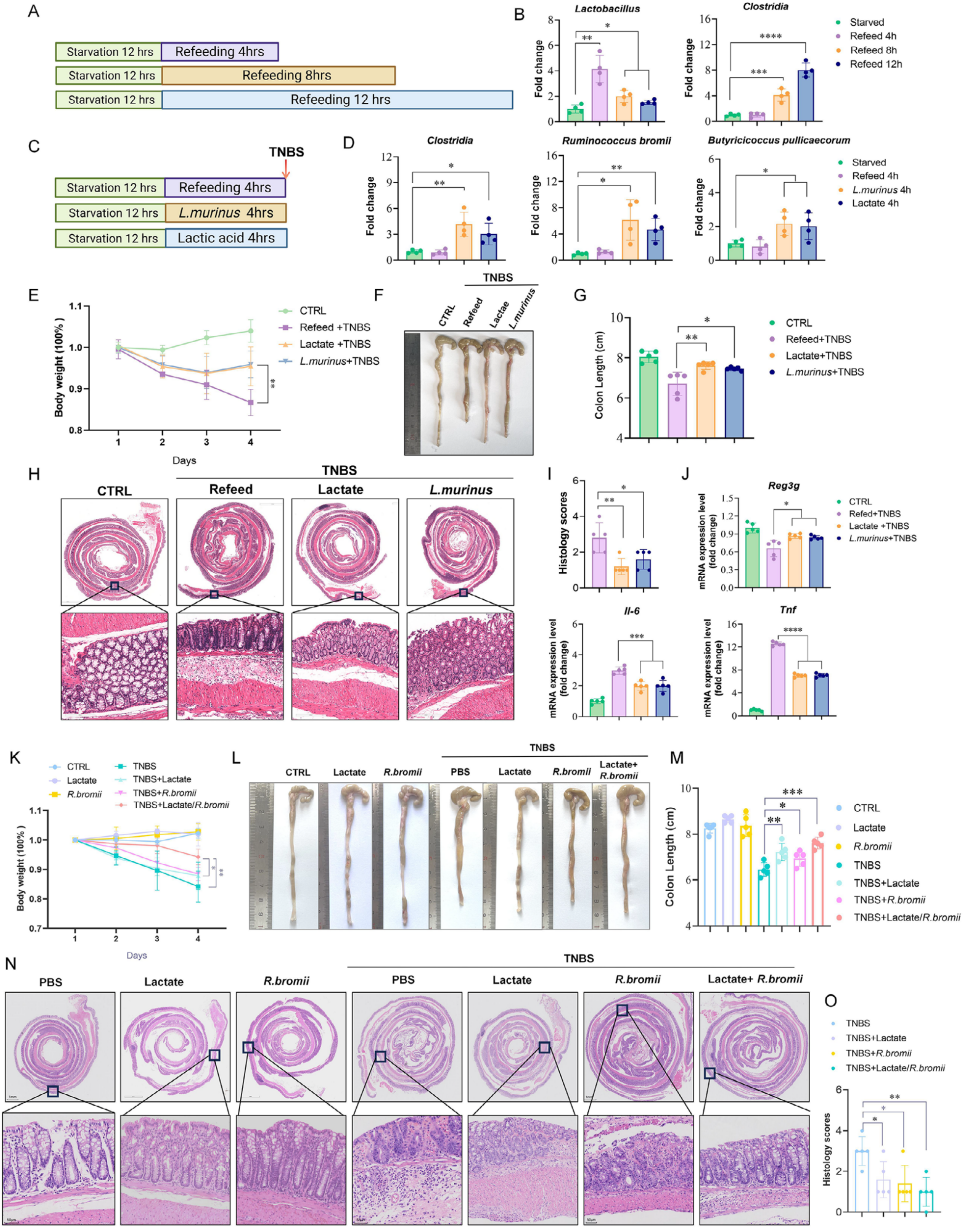
Gut lactate drives enrichment of *Ruminococcus* in TRF fasting‐feeding cycles. (A) Experimental design of the fasting and refeeding process. (B) Quantitative PCR analysis of *Lactobacillus* and *Clostridia* abundance in mouse feces during fasting‐refeeding cycles (n = 4 mice per group). (C) Experimental design involved *Lactobacillus murinus* (*L.murinus*) and lactate administration in mice during fasting‐refeeding cycles. Afterward, stool samples were collected for bacterial quantification, and these mice were challenged with TNBS to induce acute colitis. (D) Quantitative PCR analysis of *Clostridia*/*Ruminococcus*/*Butyricicoccus* levels in mice during fasting‐refeeding cycles (n = 4 mice per group). (E) Body weight changes were monitored after TNBS treatment. (F, G) Representative image of the colon and the quantitative result of colon length (n = 5 mice). (H, I) Representative images of H&E‐stained colonic tissues (scale bar: 200 µm) and pathological scores (n = 5 mice per group). (J) Relative mRNA expression of barrier protective genes *(Reg3g, Il6, and Tnf)* was measured using qRT‐PCR (n = 5 mice per group). (K) Body weight changes were monitored throughout the experimental period. (L, M) Representative image of the colon and the quantitative result of colon length (n = 5 mice). (N, O) Representative images of H&E‐stained colonic tissues (scale bar: 200 µm) and pathological scores (n = 5 mice per group). Data: Graphs represent mean ± SD. Comparisons were made by two‐tailed *t*‐test and one‐way ANOVA; *p < 0.05, **p < 0.01, ***p < 0.001, ****p < 0.0001. Data were combined from three independent experiments.

To determine whether gut lactate accumulation directly enhances *Ruminococcus* expansion, mice underwent controlled starvation followed by refeeding for 4, 8, and 12 h (Figure 8A). As anticipated, *Lactobacillus* expansion consistently preceded *Ruminococcus* enrichment, indicating a metabolic succession during refeeding (Figure 8B). Administration of *Lactobacillus murinus (L.murinus*) or exogenous lactate significantly increased *Clostridia* abundance, including *Ruminococcus bromii* (*R. bromii*) and *Butyricicoccus pullicaecorum* (Figure 8C,D), and ameliorated TNBS‐colitis, as evidenced by improved colon length and reduced histological scores compared to untreated starved mice (Figure 8E–I). These interventions also upregulated Reg3g expression while suppressing pro‐inflammatory gene expression (Figure 8J). Antibiotic‐mediated microbiota depletion reduced fecal lactate levels and *R.bromii* abundance, both of which were partially restored by lactate supplementation (Figure S18A–C). Mice with enhanced *R.bromii* colonization exhibited reduced susceptibility to TNBS‐induced colitis (Figure S18D–H), and co‐administration of lactate and *R.bromii* synergistically enhanced protection against TNBS‐induced colitis (Figure 8K–O). Collectively, these findings compellingly demonstrate that gut‐derived lactate participates in SCFA‐producing *Ruminococcus* expansion, thereby attenuating intestinal inflammation in CD models.

### 3.7 Histone Lactylation Upregulates SLC9A3 to Drive *Ruminococcus* Enrichment

**FIGURE 9:**
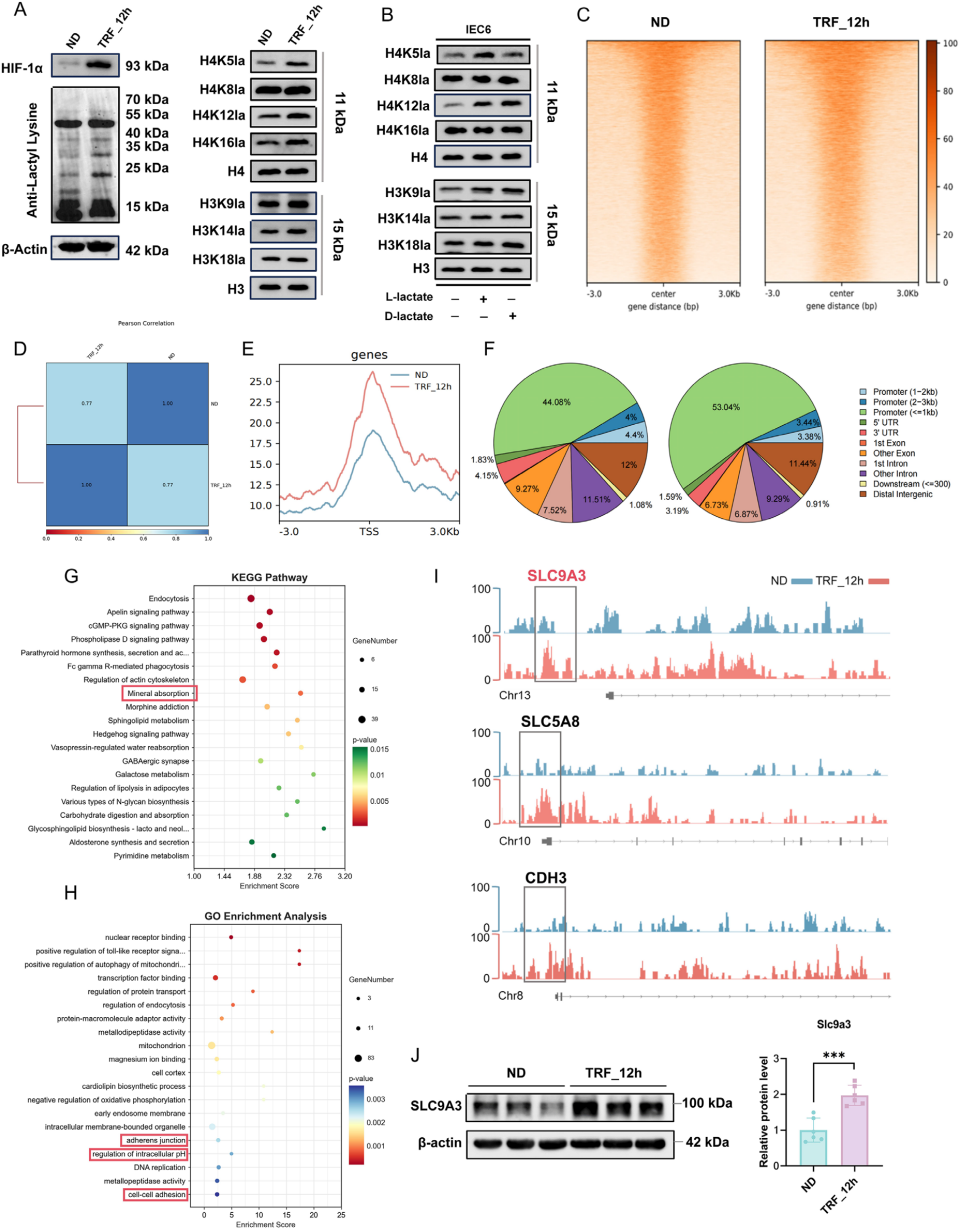
Lactate‐histone lactylation augments SLC9A3 expression to boost *Ruminococcus*. (A) Immunoblot of pan‐lactylation and histone lactylation in colonic tissues of mice receiving TRF_12h regimens and ND. (B) Immunoblot of histone lactylation levels in IEC‐6 cells treated with lactate. (C) CUT&Tag peak heatmap demonstrating binding loci in colonic tissues with TRF_12h treatment or ND group using H4K12la antibody. (D–F) The CUT&Tag method was utilized to detect the distribution of peak differences in the colonic genome following treatment with TRF or ND regimens. (G, H) KEGG pathway analysis and GO biological process analysis of regions enriched in colon following the treatment of TRF_12h. (I) IGV tracks demonstrating H4K12la CUT&Tag at *SLC9A3*, *SLC5A8*, and *Cdh3* gene locus between the control and the TRF_12h group. (J) Immunoblotting analysis of *SLC9A3* expression in the colonic tissues of mice that received TRF_12h regimens and ND (with 6 mice per group), along with the quantitative results. Data: Graphs represent mean ± SEM. Comparisons were made by two‐tailed *t*‐test and one‐way ANOVA; *p < 0.05, **p < 0.01, ***p < 0.001, ****p < 0.0001. Data were combined from three independent experiments.

We subsequently examined histone lactylation as a molecular mechanism linking lactate to microbial remodeling. Immunoblotting results demonstrated elevated pan‐lactylation and lactylated histones (H4K12la and H4K5la) in TRF_12h‐fed colons (Figure 9A). While H4K5la may also be responsive to lactate, we focused on H4K12la as it was robustly induced by D‐lactate, the predominant lactate isomer produced by gut microbiota (Figure 9B).

To screen target genes of histone lactylation in TRF, the colonic genome following treatment with TRF or ND regimens was subjected to a CUT&Tag assay using anti‐H4K12la antibody. H4K12la was enriched in promoter regions and upstream regions of several genes (Figure 9C,D; Figure S19A,B). In the TRF‐treated group, there was a noticeable increase in the transcription start site (TSS) region (Figure 9E). Analysis of the genome‐wide distribution of H4K12la demonstrated that histone modifications were located mainly within promoter regions (≤ 1 kb) (Figure 9F). KEGG pathway enrichment analysis revealed that H4K12la‐modified genes were mostly involved in endocytosis and mineral absorption, whereas GO biological process analysis indicated genes involved in adherens junction and regulation of intracellular pH (Figure 9G,H). Notably, distinct lactylation peaks were observed at the promoter region of *SLC9A3, SLC5A8*, and *CDH3* genes (Figure 9I). The *SLC9A3* represents a sodium/hydrogen exchanger that critically regulates extracellular pH and promotes anaerobic respiration [39]. Its activation acidifies the local microenvironment, creating ideal conditions for acid‐tolerant anaerobes like *Ruminococcus* [40]. Prior studies indicate that *SLC9A3* deletion in mice substantially diminishes the abundance of SCFA‐producers, including *Ruminococcaceae* and *Lachnospiraceae* [41]. Consistent with these findings, TRF_12h upregulated SLC9A3 protein in the colon (Figure 9J). Conversely, treatment with the *SLC9A3* inhibitor, Tenapanor, significantly reduced the enrichment of *R. bromii* and the subsequent barrier protection in TNBS‐induced colitis under the TRF_12h regimen (Figure S20A–F).

To establish a direct causal link between histone lactylation and SLC9A3 gene expression, a histone lactylation inhibitor, oxamate (OXA), was administered to TRF_12h‐fed mice. As shown in Figure S21A,B, TRF significantly increased colonic SLC9A3 expression, which was effectively reduced by administration of OXA. Concomitantly, inhibition of SLC9A3 abrogated the TRF‐induced *Ruminococcus* enrichment and improvement in colitis symptoms (Figure S21C–F). Histological analysis further revealed that the protective effect of TRF against TNBS‐induced colonic tissue damage was largely diminished in TRF mice treated with OXA (Figure S21G,H). These results collectively demonstrate that disrupting histone lactylation blocks the anti‐inflammatory benefits of TRF, providing direct evidence for the causal role of lactate in mediating TRF's protective effects through modulation of lactylation.

## 4 Discussion

TRF intervention has emerged as a promising dietary strategy to modulate gut microbiota composition and host metabolism [25, 42]. In this study, we identify a coordinated metabolic‐epigenetic‐microbiota axis through which a 12/12‐h fasting/feeding TRF regimen prevents and ameliorates CD‐like colitis. Mechanistically, TRF promotes the expansion of lactate‐producing *Lactobacillus*, which in turn drives enrichment of SCFA‐producing *Ruminococcus* via lactate‐induced histone H4K12 lactylation and subsequent upregulation of the sodium/hydrogen exchanger SLC9A3. The resulting *Ruminococcus*‐derived SCFAs activate epithelial HIF‐1α signaling, thereby strengthening barrier integrity and suppressing intestinal inflammation and fibrosis (Figure S22). These findings establish a mechanistic framework linking dietary timing to microbial ecology and host protection, with potential implications for CD management.

A key finding of this study is the specificity of the 12/12‐h TRF regimen, as shorter feeding windows (16/8 or 20/4 h) failed to confer comparable benefits, underscoring the importance of sufficient feeding duration to support microbial cross‐feeding interactions. The protection observed in both chemically induced (TNBS) and spontaneous (IL‐10‐KO) colitis models highlights the translational potential of this regimen. However, dietary composition—particularly fermentable fiber content—may influence microbial metabolic output and thus modulate the optimal TRF window. Future studies should systematically evaluate dietary patterns in combination with feeding‐fasting cycles to refine TRF protocols for clinical application. Microbiota transfer experiments confirmed that TRF_12h‐shaped microbiota, enriched in SCFA‐producing *Ruminococcus*, were sufficient to transfer protection. Notably, TRF enhances epithelial β‐oxidation of SCFAs while activating HIF‐1α signaling. Alterations in intestinal transit time and microbial metabolic shifts under TRF may contribute to the reduction in fecal SCFA concentrations. The observed reduction in fecal SCFA concentrations may reflect increased host uptake rather than decreased microbial production, a hypothesis that warrants investigation using isotope‐labeled tracers.

Our results identify lactate as a central orchestrator of TRF‐induced microbial remodeling. Beyond its role as a metabolic intermediate, lactate functions as an epigenetic modulator via histone lactylation [18]. We uncovered a previously unrecognized lactate‐H4K12la‐SLC9A3 signaling axis that promotes *Ruminococcus* enrichment. Specifically, *Lactobacillus*‐derived lactate induces H4K12 lactylation at the SLC9A3 promoter, upregulating this pH regulator and creating an acidic luminal niche favorable for *Ruminococcus* growth. The association between H4K12la enrichment at the SLC9A3 promoter (detected by CUT&Tag) and increased SLC9A3 expression, along with the loss of TRF effects upon inhibition of lactylation or SLC9A3 activity, strongly suggests that H4K12la contributes to SLC9A3 transcriptional upregulation. While definitive proof of direct transcriptional activation would require targeted approaches such as chromatin immunoprecipitation‐qPCR coupled with a luciferase reporter assay, our findings point to a critical role for this axis. Interestingly, fecal lactate levels did not vary linearly with fasting duration, but rather reflected a complex interplay between production, consumption, and feeding window length. In the TRF_12h group, the balanced cycle likely supports a sustained lactate pool to drive histone lactylation, explaining the observed metabolic succession wherein *Lactobacillus* expansion precedes *Ruminococcus* enrichment. While we focused on H4K12la, other lactylation marks (H4K5la) were also elevated and may play complementary roles in epithelial regeneration and immune tolerance, warranting future exploration.

Epithelial HIF‐1α activation emerged as a critical downstream mediator of TRF‐induced protection. *Ruminococcus*‐derived SCFAs, particularly butyrate, stabilized HIF‐1α and upregulated target genes involved in barrier function (MUC1, REG3G) and antimicrobial defense, while suppressing ROS‐generating enzymes (DUOX2/DUOXA2). IEC‐specific HIF‐1α knockout completely abolished TRF‐mediated protection and exacerbated colitis and fibrosis, confirming the essential role of this pathway. Notably, TRF not only attenuated inflammation but also promoted epithelial regeneration, as evidenced by increased proliferation markers (PCNA, Ki67). This regenerative effect likely results from combined SCFA‐driven metabolic support and resolution of the inflammatory microenvironment, as suggested by scRNA‐seq data showing reduced myeloid cell infiltration and downregulated chemokine signaling. Future studies using immune‐deficient models or clodronate liposomes depletion could further dissect the contributions of specific immune cell subsets in TRF‐mediated protection. Nonetheless, the present data establish IECs as central effectors of TRF, with HIF‐1α serving as a key node integrating microbial and metabolic cues.

Collectively, our study has several translational implications. First, the specificity of the 12/12‐h regimen provides a practical guideline for designing TRF trials in patients with CD. TRF may serve as an adjunctive dietary strategy for mild‐to‐moderate disease or remission maintenance, although individualization based on disease activity and nutritional status will be essential. Future clinical studies should evaluate TRF_12h in well‐phenotyped CD cohorts at risk of recurrence, assessing its impact on microbiota composition, metabolic parameters, and clinical outcomes such as mucosal healing. Second, timed administration of lactate‐producing probiotics (such as *Lactobacillus spp*.) during refeeding windows may enhance TRF benefits. Finally, the lactate‐H4K12la‐SLC9A3 signaling axis represents a novel therapeutic target, raising the possibility that pharmacologic modulation of histone lactylation could recapitulate key benefits of TRF.

## 5 Conclusion

In conclusion, this study establishes that TRF protects against CD‐like colitis through a lactate‐driven epigenetic mechanism that enriches *Ruminococcus* and activates epithelial HIF‐1α signaling. The identification of the lactate‐H4K12la‐SLC9A3 axis provides a mechanistic rationale for timed dietary interventions in CD and opens new avenues for microbiota‐targeted therapies.

## Author Contributions

C.H., H.Z., and W.Z. conceived the studies; C.H., H.Z., W.Z., L.H., and H.T. designed the studies; L.H., H.T., S.W., Y.L., L.S., S.C., Z.L., and Y.S. performed the animal experiments; C.H. and L.H. wrote the manuscript; C.H., L.H., H.T., J.C., F.T., J.W., J.H., and C.Z. performed molecular biology experiments and cell experiments. J.H., L.C., and J.C. assisted in sample collection. C.H., H.T., and L.H. prepared the tables and figures. C.H., H.Z., W.Z., L.H., H.T., and S.W. revised the manuscript. C.H., H.Z., and W.Z. supervised the study. All authors read and approved the final manuscript.

## Funding

This work was supported by the National Natural Science Foundation of China (No. 82400635 and 82300592). The Clinical Collaboration Project of Traditional Chinese and Western Medicine for Crohn's Disease (ZDYN‐2024‐A‐079). Jointly funded project by the Guangzhou Science and Technology Bureau and the Municipal Institute (2024A03J0742). The State Key Laboratory of Dampness Syndrome of Chinese Medicine by Guangdong provincial and the Ministry of China (SZ2021ZZ2702); the Sub‐Project of the National Natural Science Foundation of China Integrated Project (U23A6012). The Key Laboratory of Clinical Research on Traditional Chinese Medicine Syndromes in Guangdong Province (YN2023ZH11). The National Key Laboratory of Traditional Chinese Medicine Syndromes (QZ2025ZZ51 and QZ2025ZZ47). The Science and Technology Planning Project of Guangdong Province (2023B1212060063).

## Consent

The author has nothing to report.

## Conflicts of Interest

The authors declare no conflicts of interest.

## Supporting information




**Supporting File 1**: advs75028‐sup‐0001‐SuppMat.pdf.

**Supporting File 2**: advs75028‐sup‐0002‐TableS1‐S12.pdf.

**Supporting File 3**: advs75028‐sup‐0003‐Method.pdf.

## Data Availability

The raw metagenome sequencing data of 16S rDNA, RNA‐seq reported in this paper have been deposited in the Genome Sequence Archive database (accession no. CRA029044, CRA029026, CRA037100, CRA037002 and CRA029032).
