# Supplementary material for: Gut Lactate Boosts Ruminococcus via Histone Lactylation to Mediate Time‐Restricted Feeding Protection in Crohn's Disease
Source: Adv Sci (Weinh). 2026 Apr 7;13(33):e18419. doi: 10.1002/advs.202518419 (PMC13271634; doi:10.1002/advs.202518419)
Supplement: Supplementary file 1 — Supporting File 1: advs75028‐sup‐0001‐SuppMat.pdf. [file ADVS-13-e18419-s003.pdf]

Gut Lactate Boosts *Ruminococcus* via Histone Lactylation to Mediate Time-restricted Feeding Protection in Crohn's Disease

Linwen Huang[1,†], Huishi Tan[2,†], Senhui Weng[1,†], Yuntao Liu[3,†], Lingxu Song[3], Shaoyu Cheng[1], Zelong Lin[1], Jiawei Chen[2], Fei Tan[1], Jun Wang[1], Jinke Huang[1], Linkun Cai[1], Jiwei Chai[1], Cailing Zhong[1], Yanqiang Shi[4], Wendi Zhang[5,*], Haiyan Zhang[1,3,*], Chongyang Huang[1,3,*]

[1]Department of Gastroenterology, The Second Affiliated Hospital of Guangzhou University of Chinese Medicine, Guangzhou, China.

[2]Department of Gastroenterology and Hepatology, Guangzhou Key Laboratory of Digestive Diseases, Guangzhou Digestive Disease Center, Guangzhou First People's Hospital, School of Medicine, South China University of Technology, Guangzhou, China.

[3]Guangdong Provincial Key Laboratory of Chinese Medicine for Prevention and Treatment for Refractory Chronic Diseases; State Key Laboratory of Dampness Syndrome of Chinese Medicine; State Key Laboratory of Traditional Chinese Medicine Syndrome, Guangzhou 510120, China.

[4]Institute of Dermatology and Venereology, Dermatology Hospital, Southern Medical University, Guangzhou, China.

[5]Department of Gastroenterology, Guangdong Provincial Key Laboratory of Gastroenterology, Institute of Gastroenterology of Guangdong Province, Nanfang Hospital, Southern Medical University, Guangzhou, China.

†These authors contributed equally to this study.

***Correspondence:** Wendi Zhang (windy101@smu.edu.cn), Ph.D, Department of Gastroenterology, Nanfang Hospital, Southern Medical University, Guangzhou 510515, China. Haiyan Zhang (zhanghaiyan@gzucm.edu.cn), and Chongyang Huang (hcyoung16@gzucm.edu.cn), Ph.D, Department of Gastroenterology, The Second Affiliated Hospital of Guangzhou University of Traditional Chinese Medicine, Guangzhou 510120, China.

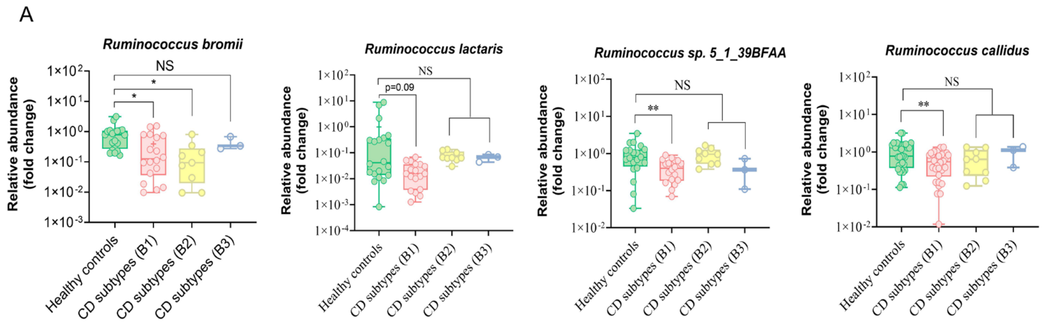

Figure S1. *Ruminococcus* levels in fecal samples from patients across all CD subtypes.

(A) Quantitative PCR detection of *Ruminococcus* species (*Ruminococcus bromii*, *Ruminococcus callidus*, *Ruminococcus lactaris*, and *Ruminococcus sp. 5_1_39BFAA*) across all CD subtypes (B1, B2, and B3) and 20 healthy controls.

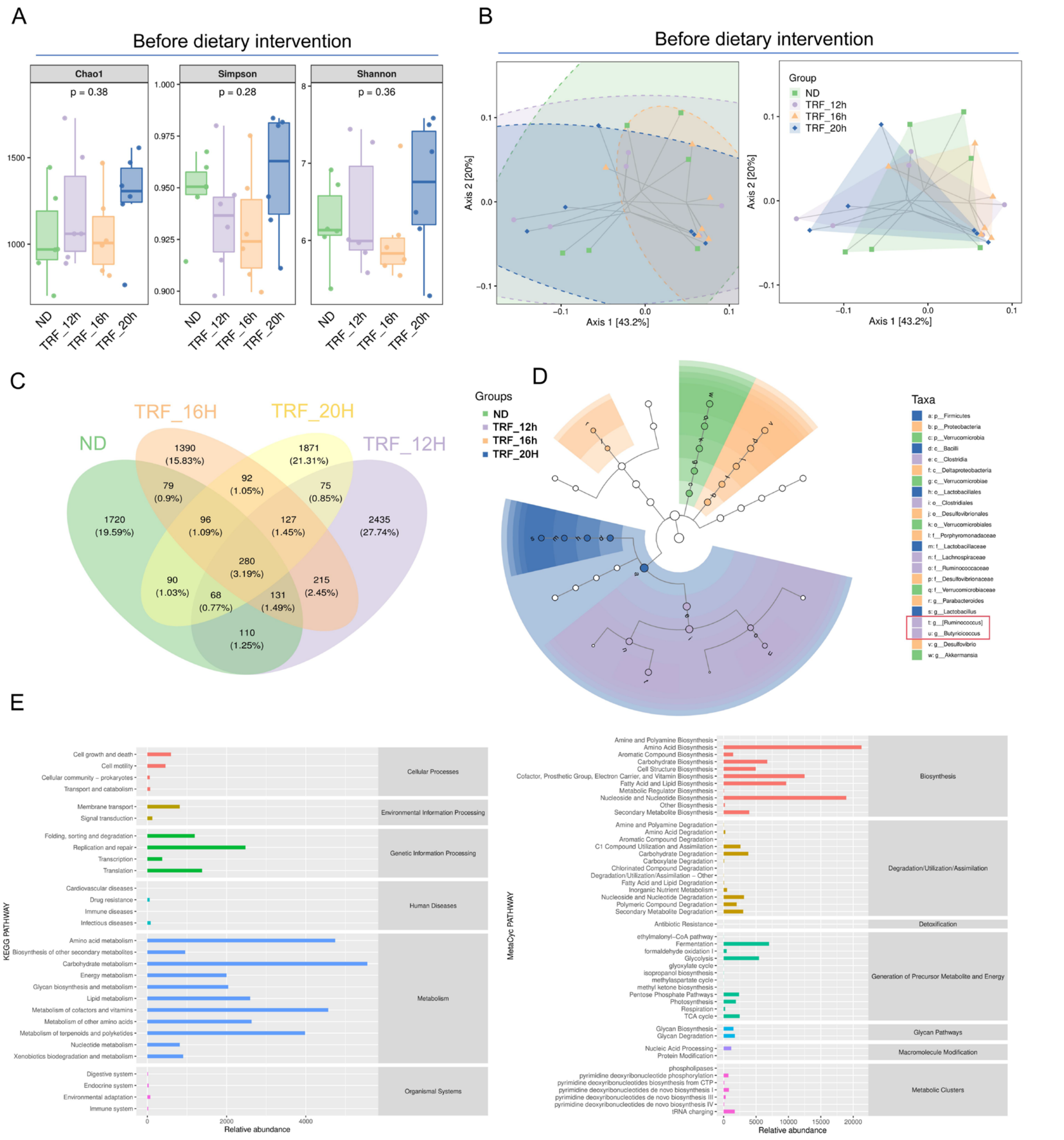

Figure S2. TRF reshapes gut microbiota composition and enriches *Ruminococcus*

(A) α-diversity analysis of gut microbes reflected by Chao1, Simpson, and Shannon before the dietary intervention. (B) PCoA analysis of fecal microbial β-diversity (Bray-Curtis distance) before the dietary intervention. (C) Venn diagram showing the overlap between the gut microbiota composition in TRF and normal diet. (D) LEfSe analysis revealing significant differences in gut microbiota composition across groups. (E) KEGG and MetaCyc pathway analysis of functional alterations in the microbiota's response to TRF regimens.

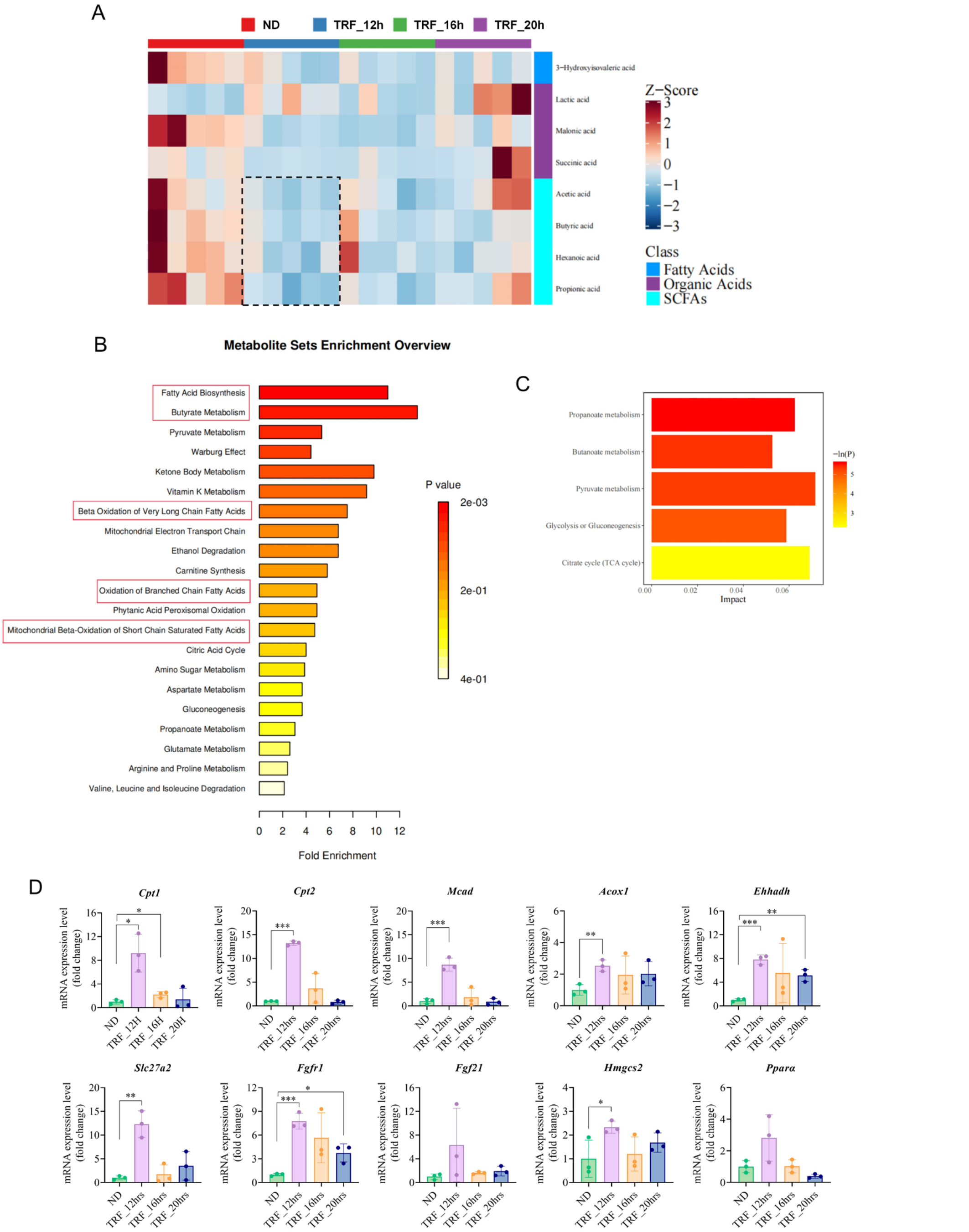

Figure S3. TRF enhances mitochondrial β-oxidation of SCFA in IECs

(A) Heatmap of fecal SCFA profiles from mice treated with different TRF cycles or ND, n=5. (B-C) The pathway enrichment analysis of differential metabolite sets in TRF_12h group. (D) qRT-PCR analysis of β-oxidation genes, including *Cpt1/2, Mcad, Acox1, Ehhadh, Slc27a2, Fgfr1, Fgf21, Ppara, Hmgcs2* (n = 3 mice).

Data: Graphs represent mean ± SD. Comparisons were made by two-tailed *t* test and one-way ANOVA; **P* < 0.05, ***P* < 0.01, ****P* < 0.001 vs. ND group. Data were combined from 3

independent experiments.

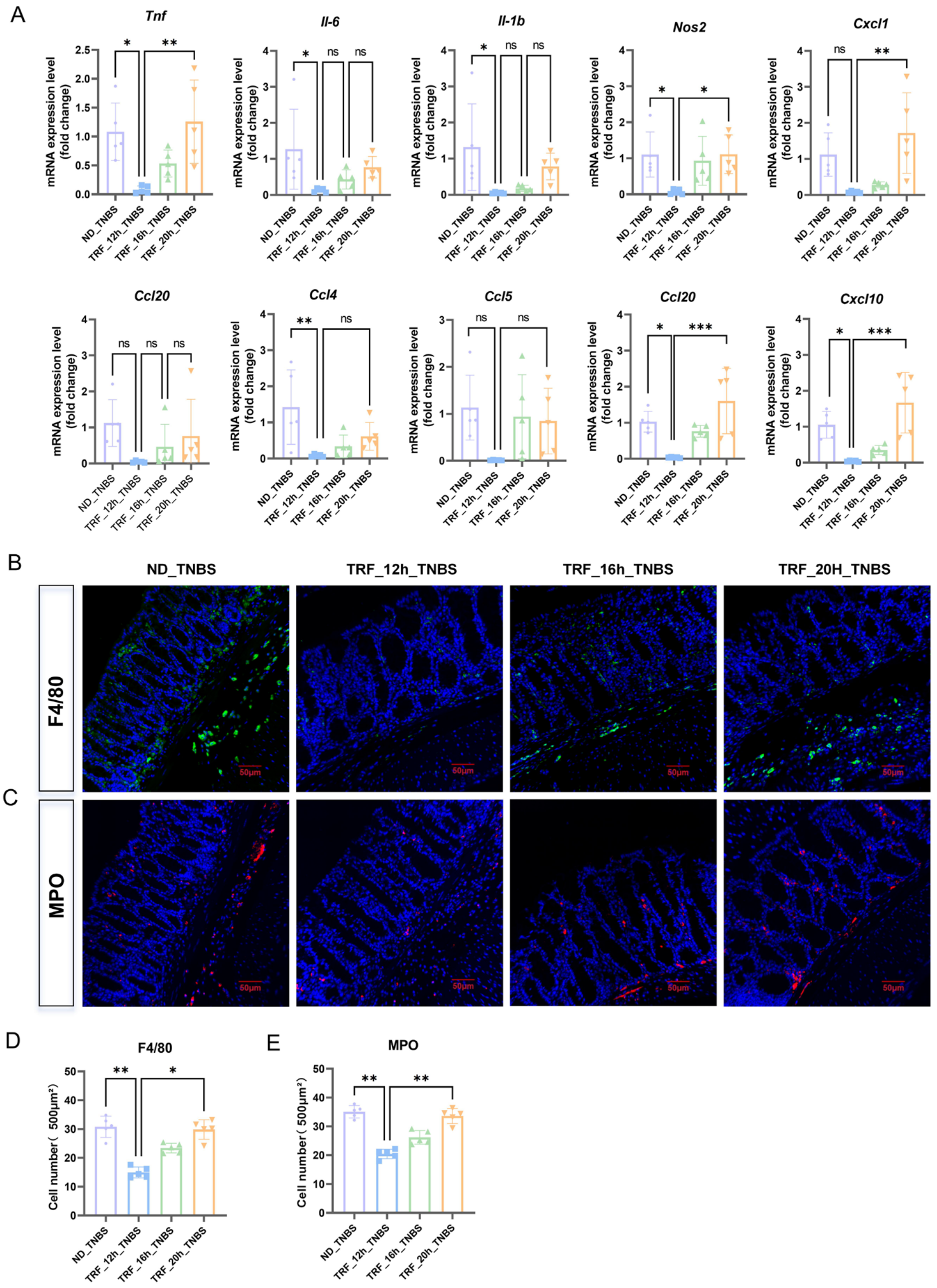

Figure S4. TRF safeguards against the TNBS-induced inflammatory response

(A) qRT-PCR analysis of pro-inflammatory genes, including *Il-6, Tnf, Nos2, Il-1b, Cxcl1, Ccl2, Ccl4, Ccl5, Cxcl10, and Ccl20* (n = 5 mice). (B-C) Representative image of F4/80 and MPO staining (green/red; scale bar: 50 μm). (D-E) Quantification of F4/80- and MPO-positive cell numbers (n=5 mice per group).

Data: Graphs represent mean ± SD. Comparisons were made by two-tailed *t* test and one-way

ANOVA; *$P < 0.05$, **$P < 0.01$, ***$P < 0.001$ vs. ND group. Data were combined from 3 independent experiments.

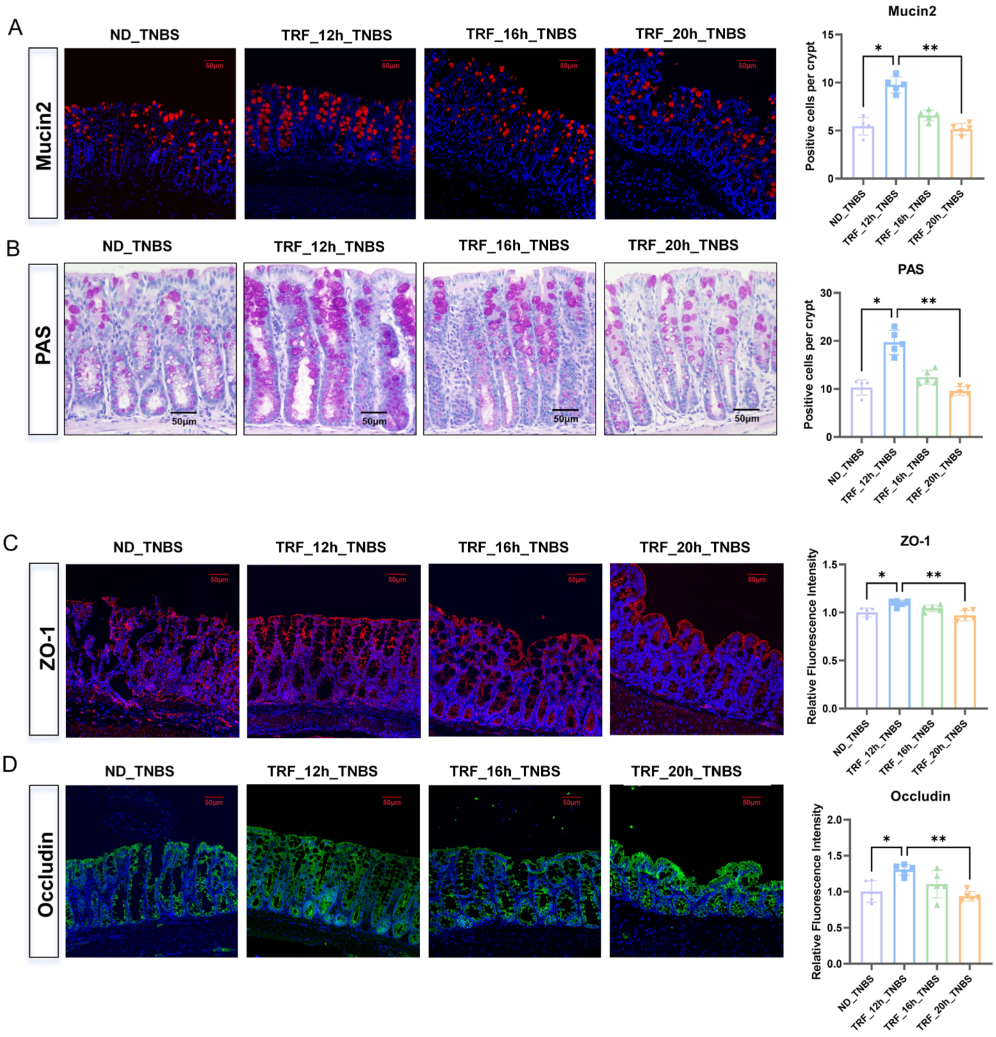

Figure S5. TRF ameliorated barrier dysfunction in TNBS-induced chronic colitis.

(A) Representative image of Mucin2 staining and quantification of Mucin2 cell numbers (red; scale bar: 50 μm). (B) Representative image of PAS staining and quantification of PAS-positive cell numbers (red/purple; scale bar: 50 μm). (C) Representative image of ZO-1 staining (red; scale bar: 50 μm) and quantification of ZO-1-positive areas. (D) Representative image of occludin (staining green; scale bar: 50 μm) and quantification of occludin-positive areas (n=5 mice per group).

Data: Graphs represent mean ± SD. Comparisons were made by two-tailed *t* test and one-way ANOVA; *$P < 0.05$, **$P < 0.01$ vs. ND_TNBS group. Data were combined from 3 independent

experiments.

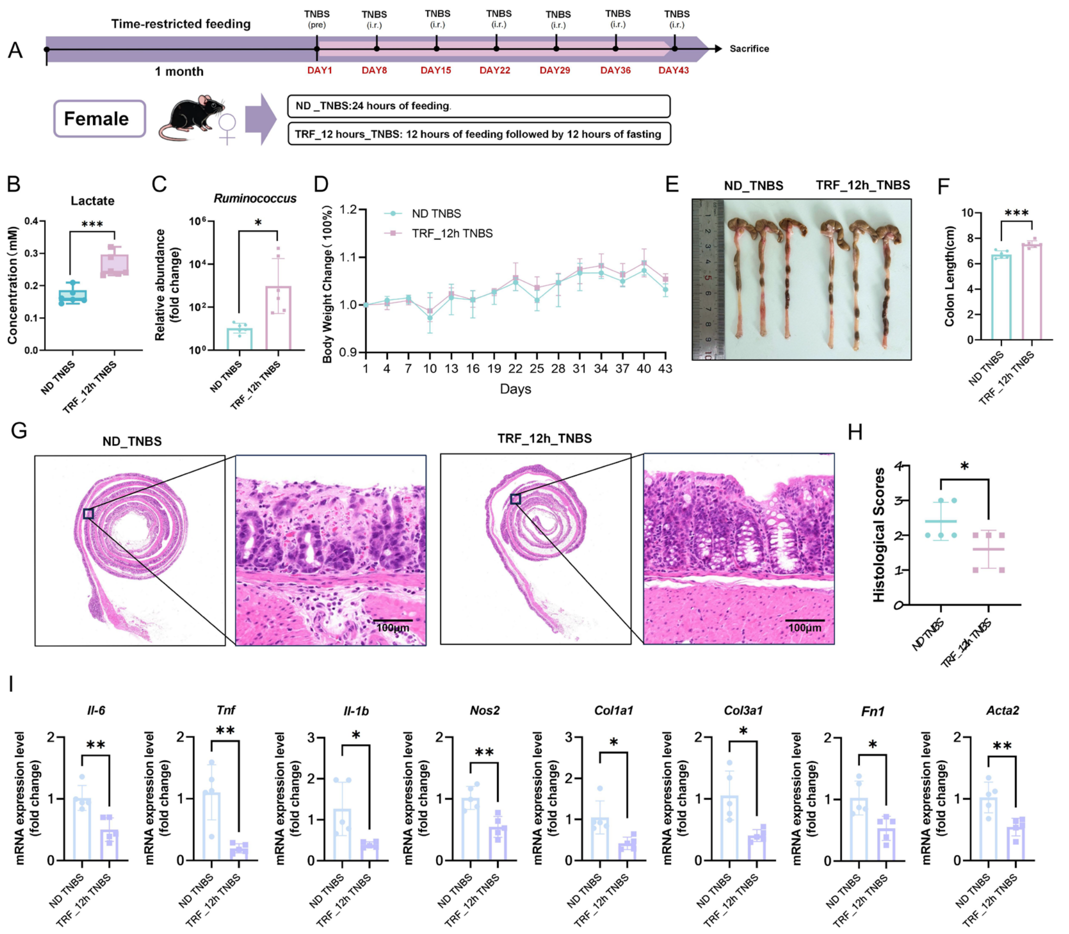

Figure S6. TRF attenuates TNBS-induced colitis severity and intestinal fibrosis in female mice

(A) The experimental design of TRF regimens (Female mice that received a normal diet and TRF diets with 12/12 fasting/feeding cycles). (B) Fecal lactate concentration in female mice subjected to a normal diet or TRF diets with 12/12 fasting/feeding cycles (n=6 mice per group). (C) Quantitative PCR analysis of *Ruminococcus bromii* levels in female mice received a normal diet and TRF diets with 12/12 fasting/feeding cycles (n=6 mice per group). (D) Body weight changes were monitored throughout the experimental period. (E-F) Colon length quantification (n=6 mice per group). (G) Representative H&E staining of colonic tissues (scale bar: 100 μm). (H) Histopathological scoring of epithelial injury and immune infiltration (n=5 mice per group). (I) The relative mRNA expression levels of pro-inflammatory (*Tnf, Il-1b, Il-6, Nos2*) and pro-fibrotic genes (*Fn1, Acta2, Col3a1, and Col1a1*) were measured using qRT-PCR.

Data: Graphs represent mean ± SEM. Comparisons were made by two-tailed *t*-test; $*P < 0.05$,

P < 0.01,*P < 0.001 vs. ND_TNBS group. Data were combined from 3 independent experiments.

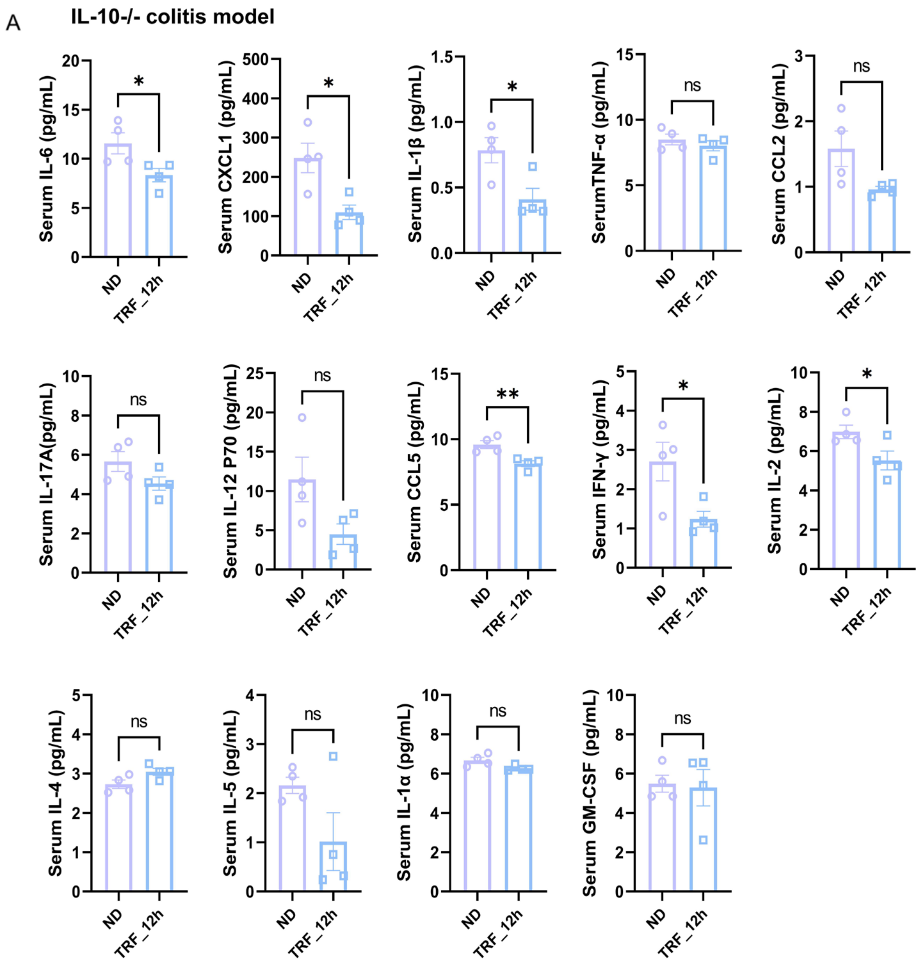

Figure S7. TRF reduced serum inflammatory cytokines in IL-10-deficient colitis

(A) Serum levels of inflammatory cytokines, such as IL-6, TNF-α, IL-1β, CXCL1, CCL2, IL-17A, IL-12p70, CCL5, IFN-γ, IL-2, IL-4, IL-5, IL-1α, and GM-CSF, in the IL-10-/- colitis model.

Data: Graphs represent mean ± SD. Comparisons were made by two-tailed *t* test; *P < 0.05, **P < 0.01 vs. ND group. Data were combined from 3 independent experiments.

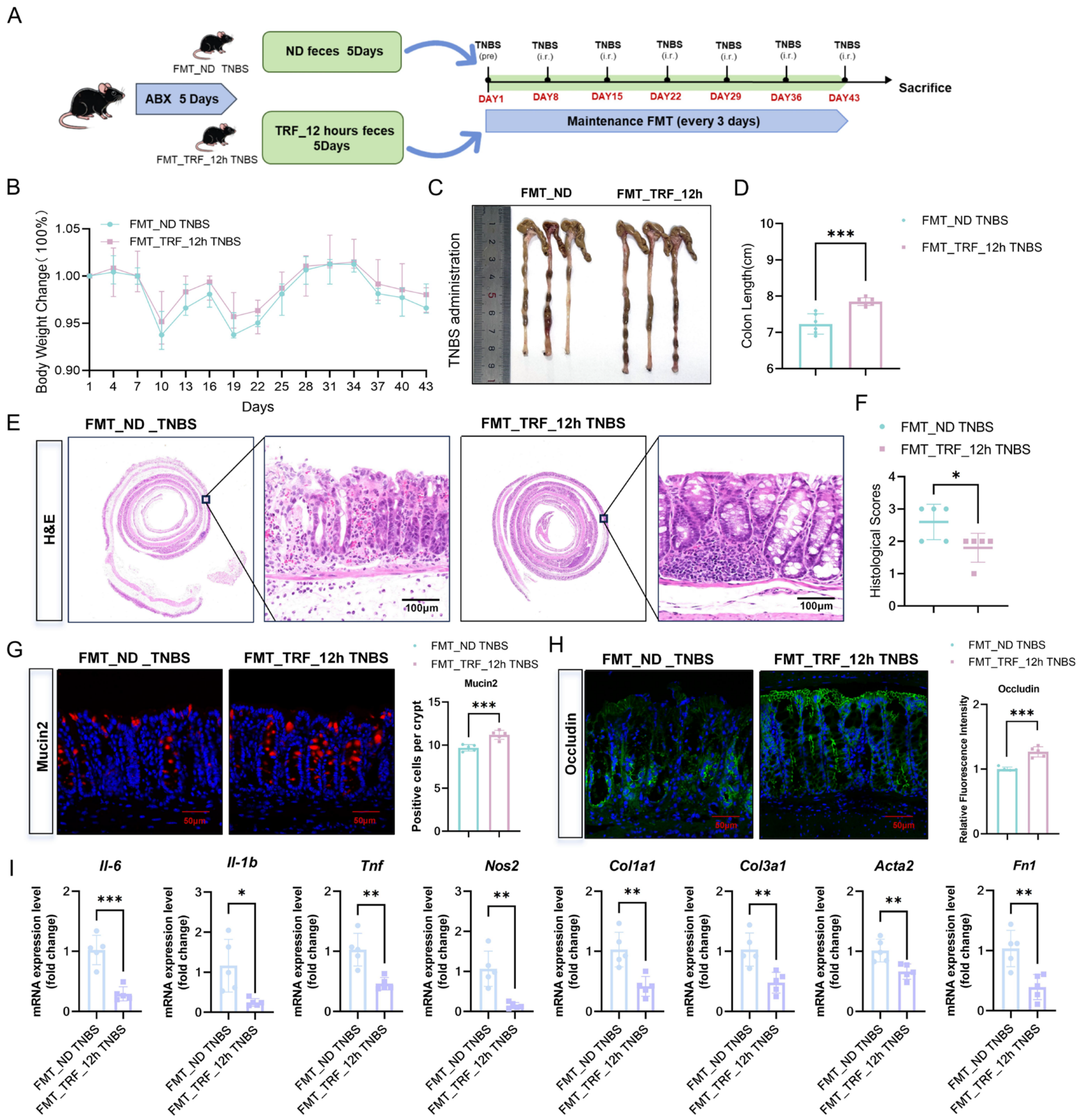

Figure S8. TRF-shaped microbiota confers protection against TNBS-induced colitis

(A) Experimental design of fecal bacterial transplantation. (B) Body weight changes were monitored throughout the experimental period. (C-D) Colon length quantification (n=6 mice per group). (E) Representative H&E staining of colonic tissues (scale bar: 100 μm). (F) Histopathological scoring of epithelial injury and immune infiltration (n=5 mice per group). (G) Representative image of Mucin2 (red; scale bar: 50 μm) and quantification of Mucin2-positive cell numbers (n=5 mice per group). (H) Representative image of occludin staining (green; scale bar: 50 μm) and quantification of occludin-positive areas (n=5 mice per group). (I) The relative mRNA expression levels of pro-inflammatory (*Tnf, Il-1b, Il-6, Nos2*) and pro-fibrotic genes (*Fn1, Acta2, Col3a1, and Col1a1*) were measured using qRT-PCR.

Data: Graphs represent mean ± SD. Comparisons were made by two-tailed *t*-test; $^{*}P < 0.05$, $^{**}P < 0.01$, $^{***}P < 0.001$. Data were combined from 3 independent experiments.

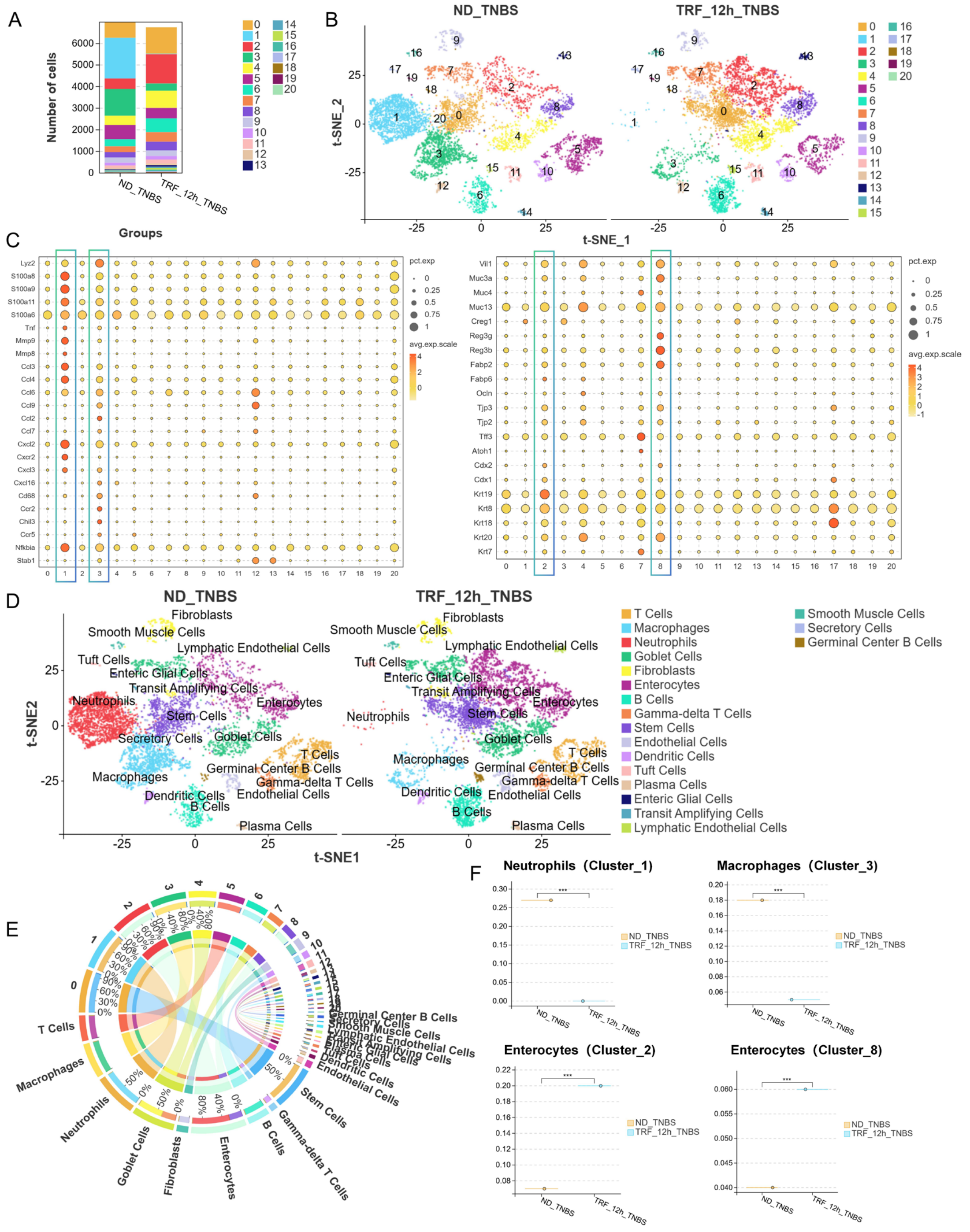

Figure S9. TRF modulates cellular composition and transcriptional landscape in TNBS-treated colonic tissue

(A) Bar plots showing the proportions of different cell subsets between ND_TNBS and TRF_TNBS groups. (B) t-SNE projection of cellular subpopulations. (C)Bubble plot visualizing the expression of selected marker genes across indicated cell subsets. (D)The t-SNE plot shows the characteristics of related cells in ND_TNBS and TRF_TNBS. (E) Circos plots depicting cell-cell interaction networks between subsets in the ND_TNBS and TRF_TNBS groups. (F) Comparison of the proportion of neutrophils (Cluster 1), macrophages (Cluster 3), enterocytes (Cluster 2) and enterocytes (Cluster 8) between the ND_TNBS and TRF groups.

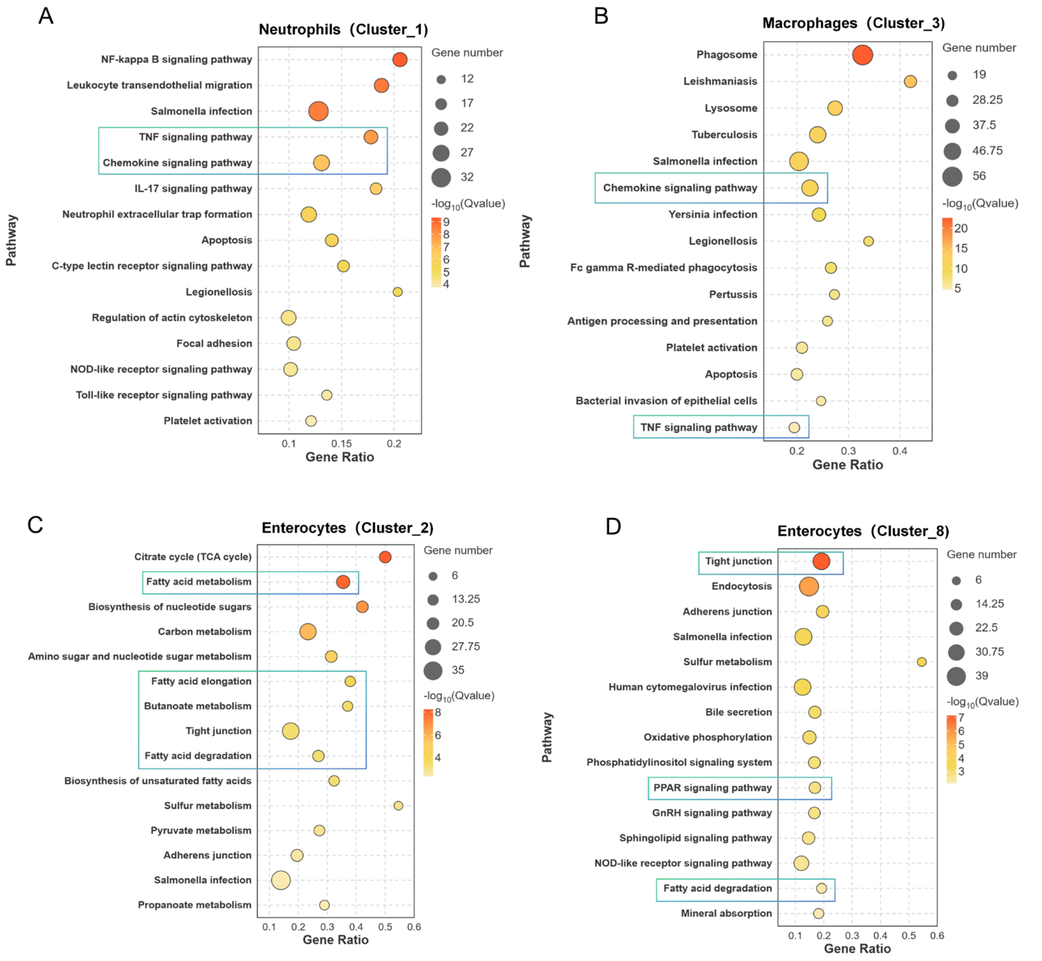

Figure S10. KEGG pathway enrichment in immune cells and enterocytes

(A) Pathways enriched in neutrophils (Cluster 1). (B) Pathways enriched in macrophages (Cluster 3). (C) Pathways enriched in enterocytes (Cluster 2). (D) Pathways enriched in enterocytes (Cluster 8).

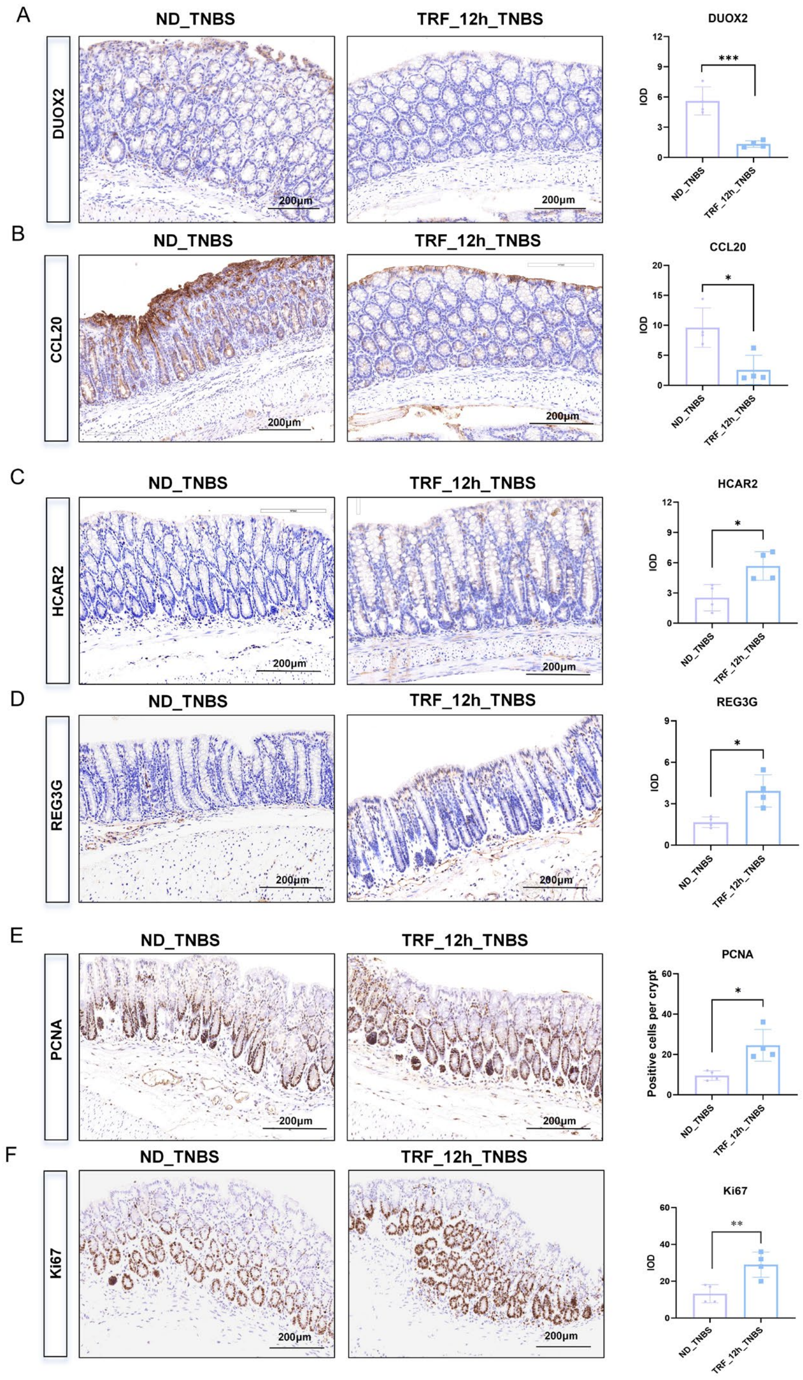

Figure S11. TRF regulates epithelial barrier integrity and tissue repair

(A-F) Representative immunohistochemical staining for DUOX2, CCL20, HCAR2, REG3G, PCNA, and Ki67 in the colonic epithelium (brown: positive signal; scale bar:200 μm). Quantitative analysis of DUOX2, CCL20, HCAR2, REG3G, PCNA, and Ki67-positive areas, respectively.

Data: Graphs represent mean ± SD. Comparisons were made by two - tailed *t* - test; *$p < 0.05$, **$p < 0.01$, ***$p < 0.001$.

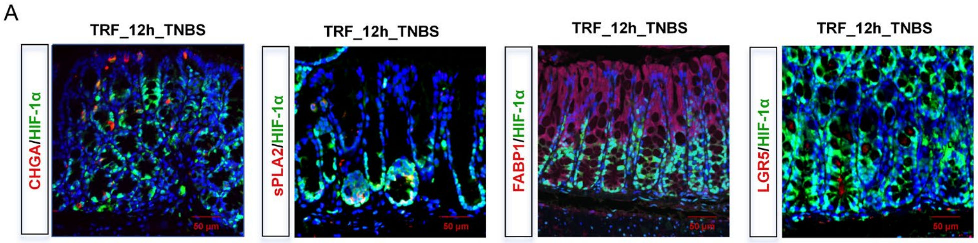

Figure S12. Detection and precise mapping of HIF-1α-expressing intestinal epithelial cells

(A) Immunofluorescence double staining of HIF-1α (green) and CHGA, sPLA2, FABP1, or LGR5 (red) (scale bars: 50 µm).

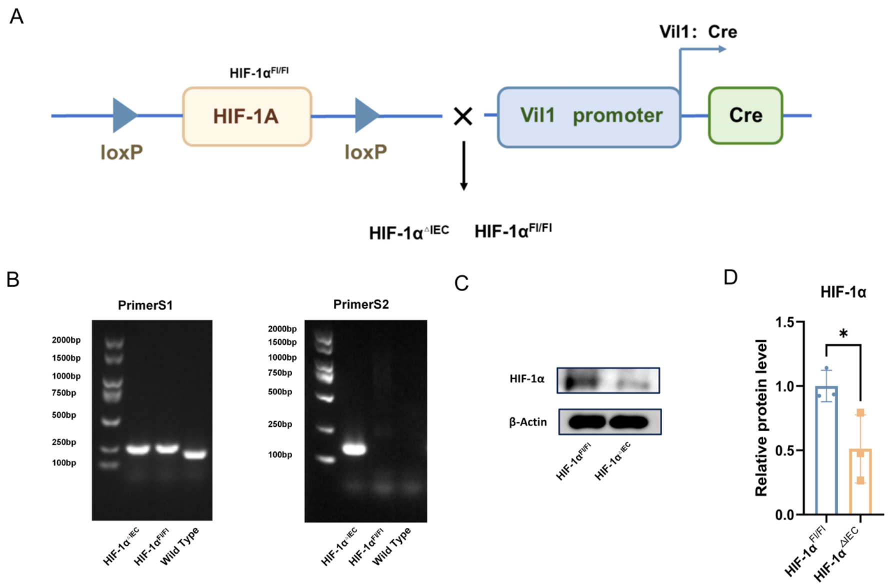

Figure S13. Generation and establishment of HIF-1α*fl/fl*Vil1-Cre mice

(A) Generation plot of intestinal epithelial cell (IECs)-specific HIF-1α gene knockout mice (HIF-1α*fl/fl*Vil1-Cre) using the Cre-loxP method. (B) Genotyping was performed using genomic DNA isolated from 8-week-old mice and primers by PCR. The PCR products were separated using agarose gel electrophoresis to identify PCR products. (C-D) Immunoblotting analysis of HIF-1α expression in Intestinal epithelial cells from HIF-1α*fl/fl* Vil1-Cre mice and their wild-type littermates.

(The data are presented as mean±SD in each group. n=3. *P <0.05 by unpaired Student's t test. Data shown are representative of three independent experiments.)

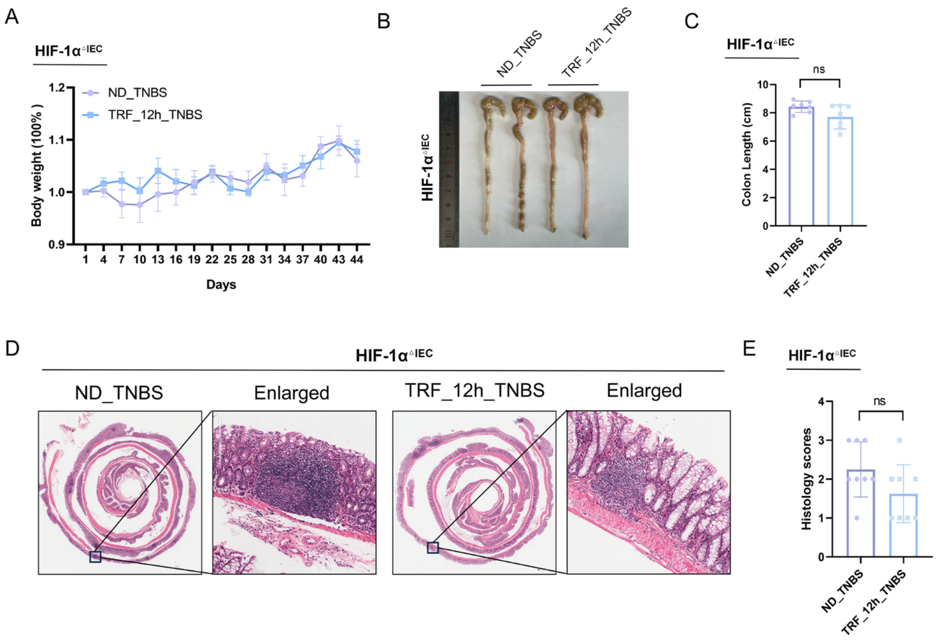

Figure S14. Deletion of epithelial HIF-1α attenuates protective effect of TRF in TNBS-induced chronic colitis

(A) Body weight changes were monitored throughout the experimental period. (B-C) Colon length quantification (n=8 mice per group). (D) Representative H&E staining of colonic tissues (scale bar: 100 μm). (E) Histopathological scoring of epithelial injury and immune infiltration (n=8 mice per group).

(The data are presented as mean ± SD in each group. n=8. ns: Non-significant; by unpaired Student's *t* test. Data shown are representative of three independent experiments.)

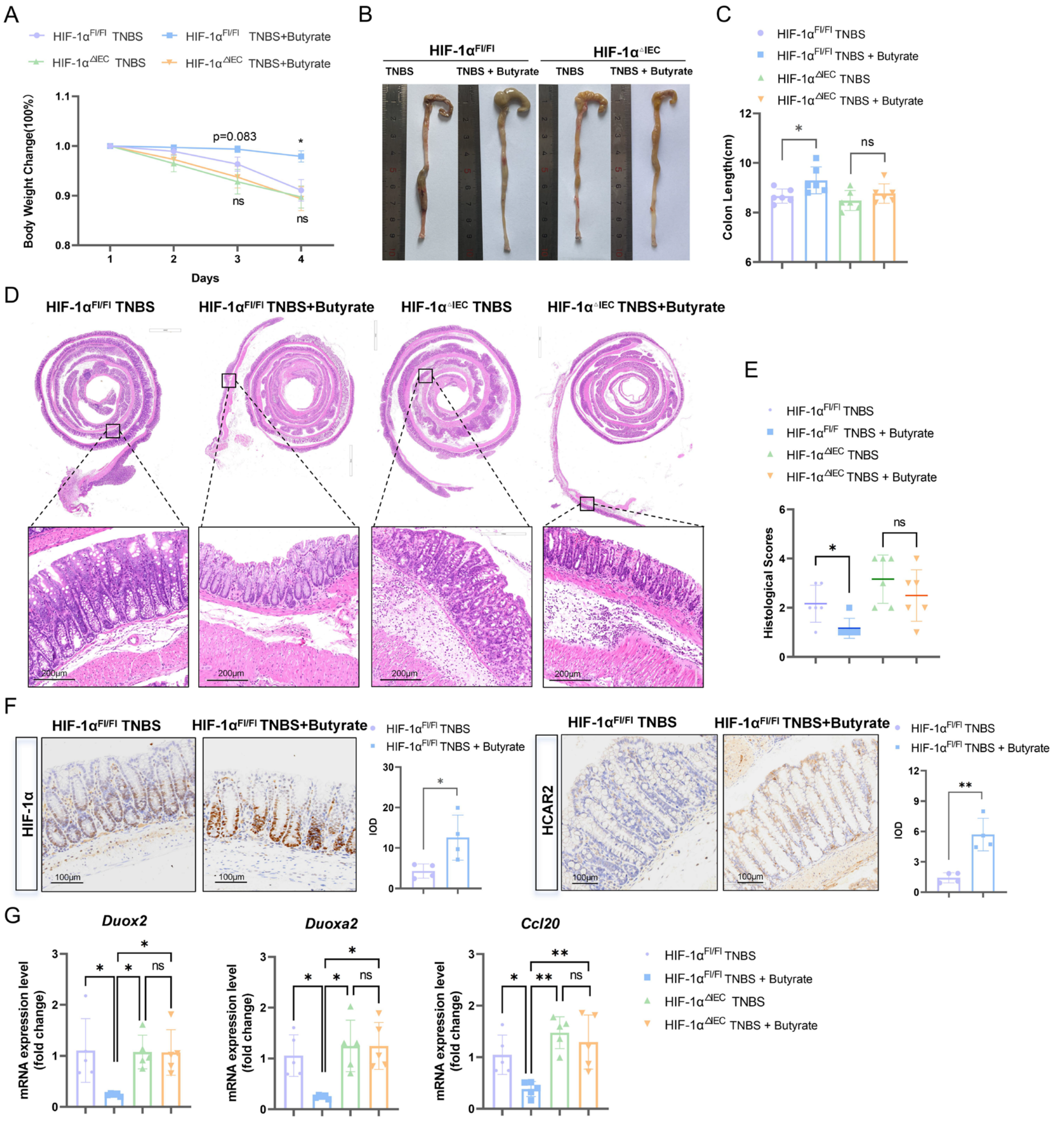

Figure S15. Epithelial HIF-1α mediates butyrate's protective role against colitis

(A) Body weight changes in butyrate-treated HIF-1α$^{\Delta IEC}$ and WT littermates subjected to TNBS-induced colitis throughout the experimental period. (B-C) Colon length quantification (n=6 mice per group). (D) Representative H&E staining of colonic tissues (scale bar: 100 μm). (E) Histopathological scoring of epithelial injury and immune infiltration (n=6 mice per group). (F) Representative immunohistochemical staining of HCAR2 and HIF-1α in the colonic epithelium (brown: positive signal; scale bar: 100μm) Quantitative analysis of HCAR2-positive and HIF-1α-positive areas, respectively. (G) The relative mRNA expression levels of *Duox2, Duoxa2 and Ccl20* Were measured using qRT-PCR.

Data: Graphs represent mean ± SD. Comparisons were made by two-tailed *t* test and one-way ANOVA; ns: Non-significant, *$P < 0.05$, **$P < 0.01$ Data were combined from 3 independent

experiments.

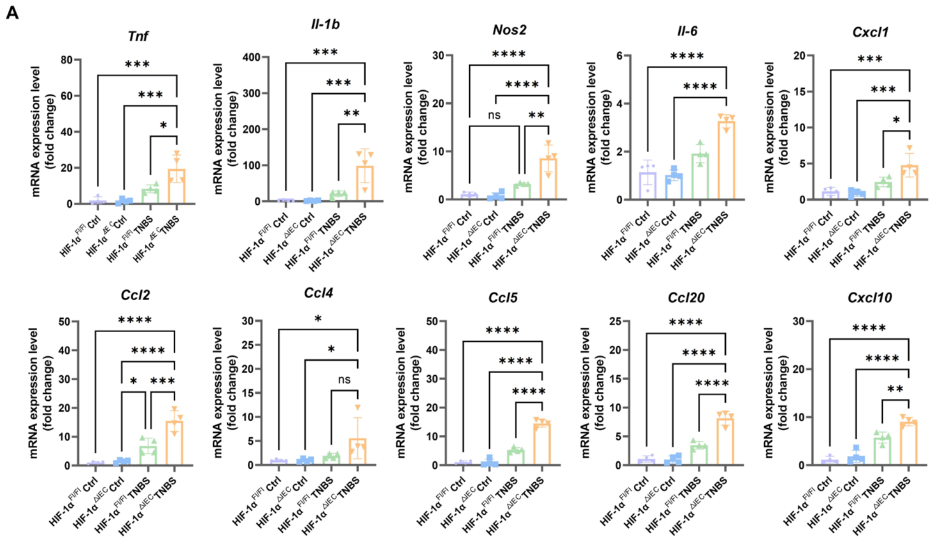

Figure S16. Deletion of epithelial HIF-1α exacerbates TNBS-induced colonic inflammation

(A) The relative mRNA expression levels of pro-inflammatory genes(*Tnf, Il-1b, Il-6, Nos2, Cxcl1, Ccl2, Ccl4, Ccl5, Ccl20, and Cxcl10*) was measured using qRT-PCR.

(The data are presented as mean ± SD in each group. n=4. **P* <0.05; ***P* <0.01; ****P* <0.001; *****P* <0.0001 by unpaired Student's *t* test. Data shown are representative of three independent experiments.)

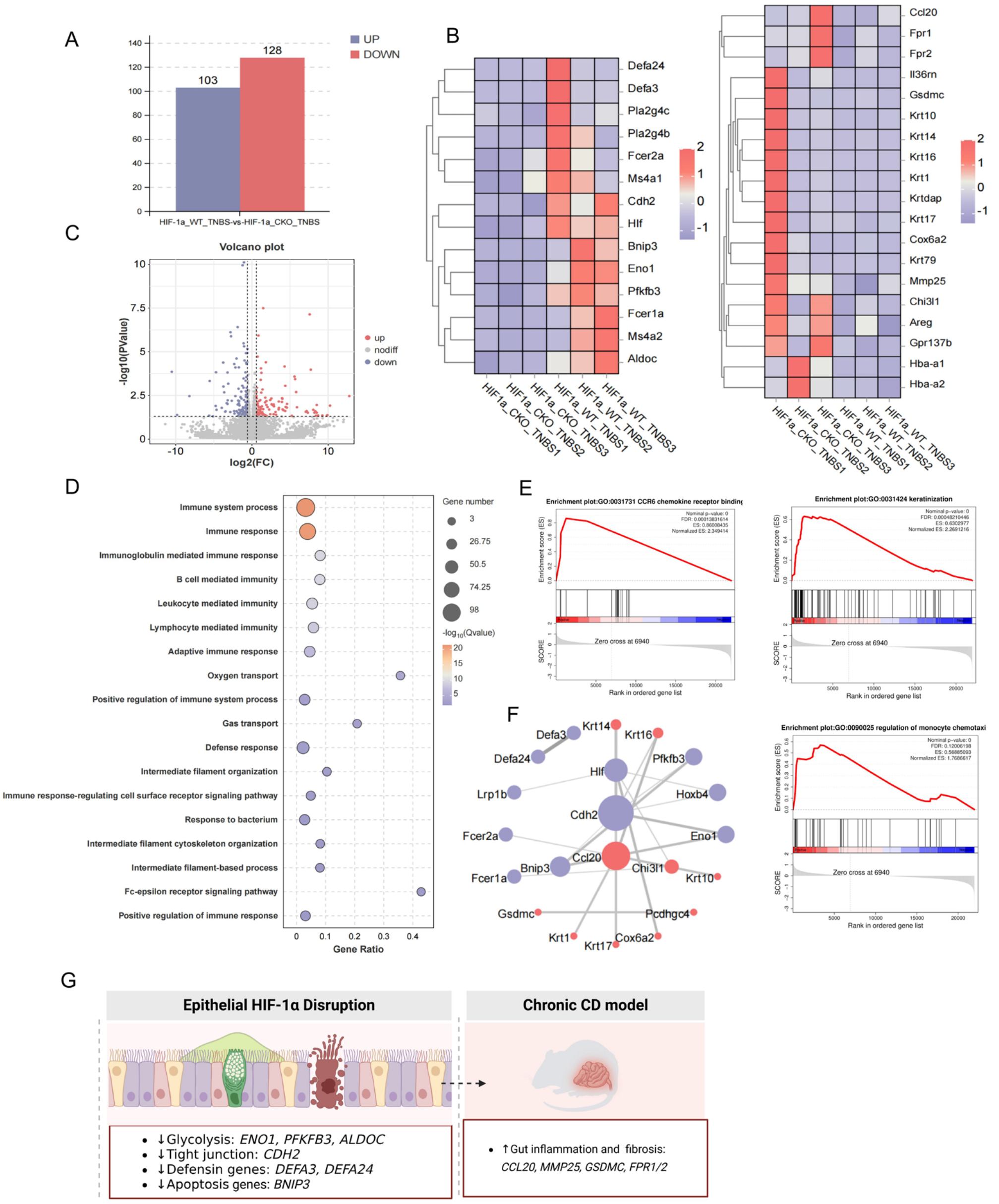

Figure S17. Epithelial HIF-1α regulates multiple pathways to protect against TNBS-induced intestinal inflammation

(A) The overall differentially gene expression numbers were displayed as a histogram. (B) Heatmap representation of differential expressed genes in colonic tissues of HIF-1α$^{fl/fl}$Vil1-Cre(CKO) and HIF-1α$^{fl/fl}$(WT) mice subjected to TNBS treatment. (C) Volcano plot showing the significantly changed genes in the TNBS-treated colon between the CKO and WT groups. (D) GO biological analysis in CKO mice compared to the WT mice group. (E) GSEA snapshots of GO pathway enrichment analysis: CCR6 chemokine receptor binding; Regulation of monocyte chemotaxis; Keratinization. (F) PPI network of differentially expressed genes. (G) Schematic diagram illustrating the potential mechanism of epithelial HIF-1α signaling in the

protection against CD intestinal inflammation.

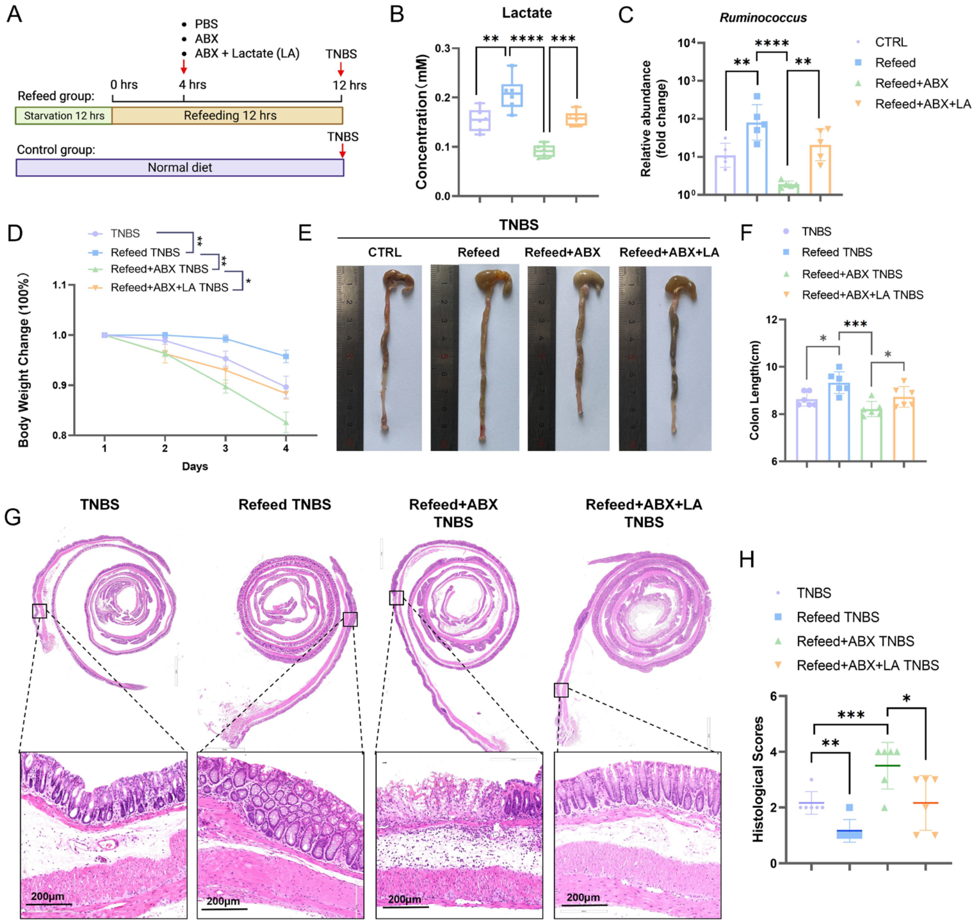

Figure S18. ***Lactobacillus*-derived lactate enriches *Ruminococcus* and alleviates colitis in TRF fasting-feeding cycles**

(A) Experimental design of exogenous lactate supplementation during the fasting-refeeding transition. (B) Fecal lactate concentration in mice during the fasting-refeeding transition. (n=6 mice per group). (C) Quantitative PCR analysis of *Ruminococcus bromii* levels in fecal samples from mice (n=5 mice per group). (D) Body weight changes were monitored throughout the experimental period. (E-F) Colon length quantification (n=6 mice per group). (G) Representative H&E staining of colonic tissues (scale bar: 200 μm). (H) Histopathological scoring of epithelial injury and immune infiltration (n=6 mice per group).

Data: Graphs represent mean ± SD. Comparisons were made by two-tailed *t* test and one-way ANOVA; ns: Non-significant, $*P < 0.05$, $**P < 0.01$, $***P < 0.001$, $****P < 0.0001$. Data were combined from 3 independent experiments.

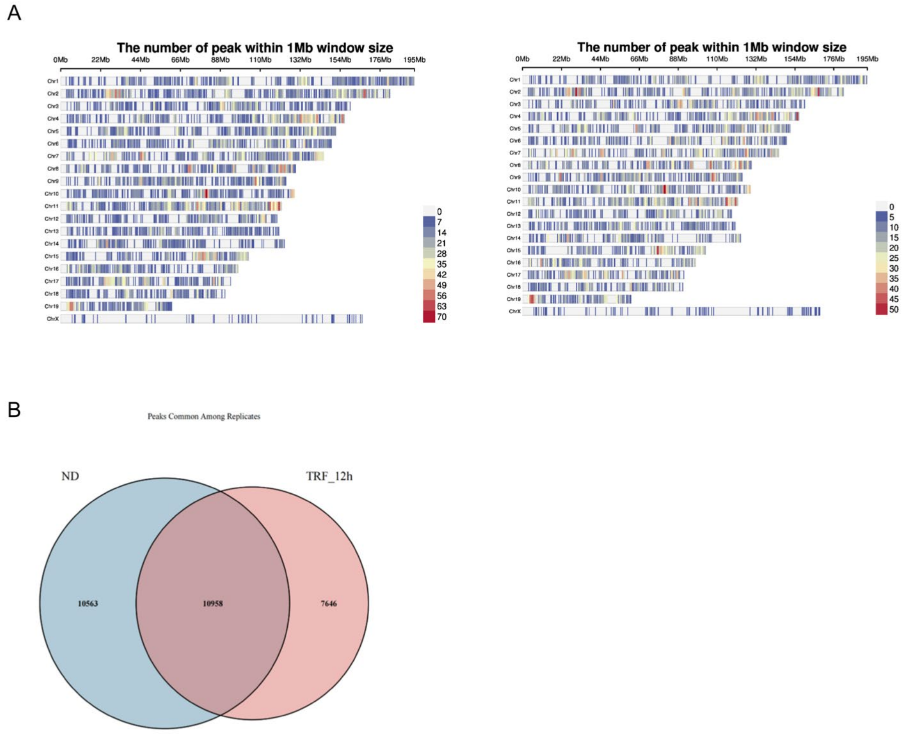

Figure S19. TRF enhances histone lactylation to regulate gene transcription

(A) The differential binding peaks of H4K12la within 1Mb window size were displayed as a histogram. (B) Venn diagram showed H4K12la peaks in TRF- and ND-treated colon.

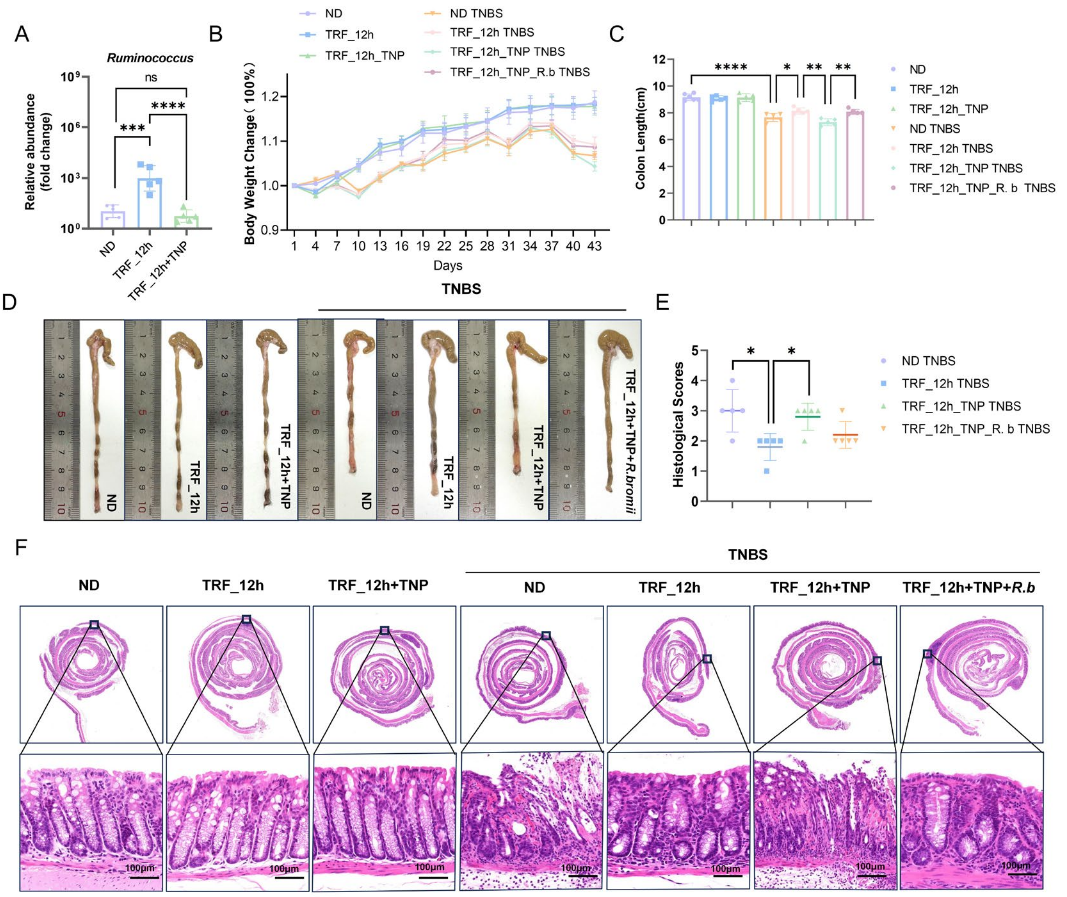

Figure S20. Inhibition of SLC9A3 abrogates the TRF-induced enrichment of *Ruminococcus* and its barrier-protective effects

(A) Quantitative PCR analysis of *Ruminococcus bromii* levels in fecal samples from mice (n=5 mice per group). (B) Body weight changes were monitored throughout the experimental period. (C-D) Colon length quantification (n=5 mice per group). (E-F) Representative H&E staining of colonic tissues (scale bar: 100 μm) and histopathological scoring of epithelial injury and immune infiltration (n=5 mice per group). Data: Graphs represent mean ± SD. Comparisons were made by two-tailed *t* test and one-way ANOVA; ns: Non-significant, **P* < 0.05, ***P* < 0.01,****P* < 0.001,**** *P* < 0.0001. Data were combined from 3 independent experiments.

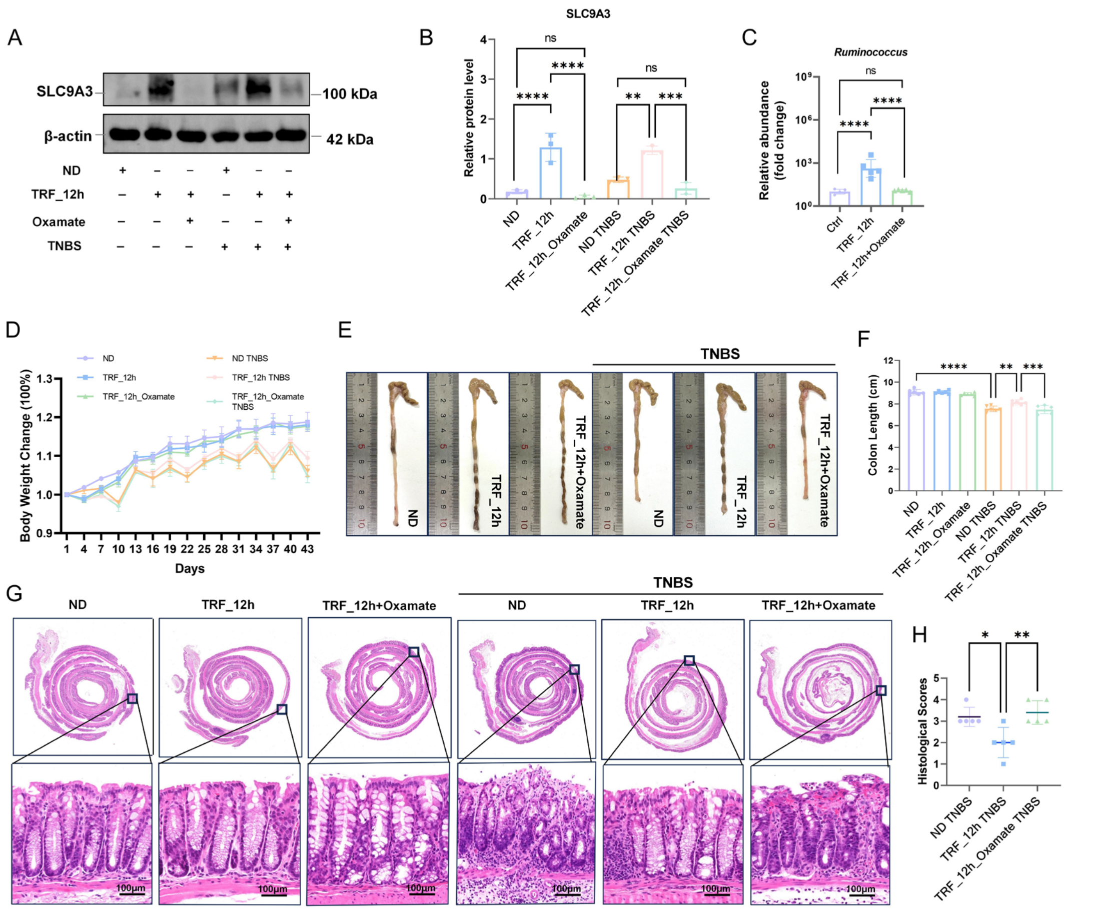

Figure S21. Oxamate treatment impairs the lactate-H4K12la-SLC9A3 axis and the therapeutic benefits of TRF

(A-B) Immunoblotting analysis of SLC9A3 expression in the colonic tissues of mice (with 3 mice per group) along with the quantitative results. (C) Quantitative PCR analysis of *Ruminococcus bromii* levels in fecal samples from mice (n=6 mice per group). (D) Body weight changes were monitored throughout the experimental period. (E-F) Colon length quantification (n=6 mice per group). (G) Representative H&E staining of colonic tissues (scale bar: 100 μm). (H) Histopathological scoring of epithelial injury and immune infiltration (n=5 mice per group). Data: Graphs represent mean ± SD. Comparisons were made by two-tailed *t* test and one-way ANOVA; ns: Non-significant, *$P < 0.05$, **$P < 0.01$,***$P < 0.001$,**** $P < 0.0001$. Data were combined from 3 independent experiments.

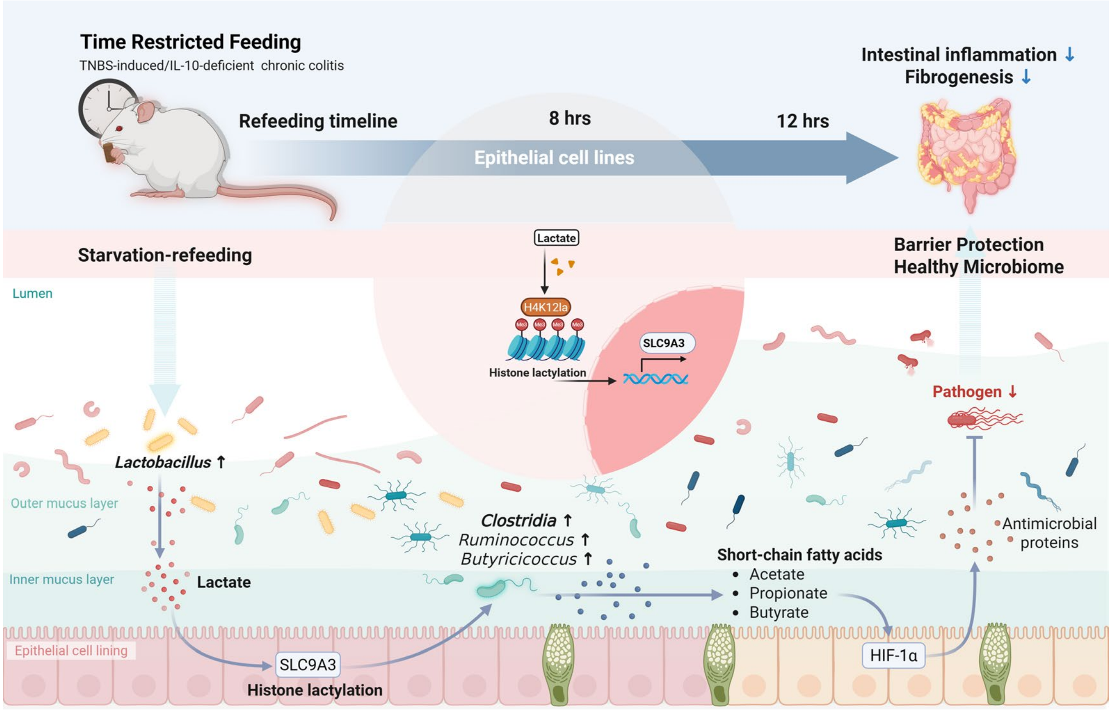

Figure S22. TRF mediates barrier protection by enriching SCFA-producing *Ruminococcus* via histone lactylation

TRF enriches *Ruminococcus*, a genus of bacteria that produces short-chain fatty acids (SCFAs), and activates the epithelial HIF-1α signaling pathway. This mechanism serves to safeguard the colonic mucosa against inflammatory insults in colitis models induced by TNBS and interleukin-10 deficiency. Mechanistically, gut lactate production during periods of starvation and refeeding mediates histone lactylation (H4K12la), which augments SLC9A3 expression and establishes an acidic gut microenvironment to support the rapid enrichment of *Ruminococcus*.
