# Supplementary material for: Gut Lactate Boosts Ruminococcus via Histone Lactylation to Mediate Time‐Restricted Feeding Protection in Crohn's Disease
Source: Adv Sci (Weinh). 2026 Apr 7;13(33):e18419. doi: 10.1002/advs.202518419 (PMC13271634; doi:10.1002/advs.202518419)
Supplement: Supplementary file 2 — Supporting File 2: advs75028‐sup‐0002‐TableS1‐S12.pdf. [file ADVS-13-e18419-s002.pdf]

# Gut Lactate Boosts *Ruminococcus* via Histone Lactylation to Mediate Time-restricted Feeding Protection in Crohn's Disease

**Linwen Huang[1,†], Huishi Tan[2,†], Senhui Weng[1,†], Yuntao Liu[3,†], Lingxu Song[3], Shaoyu Cheng[1], Zelong Lin[1], Jiawei Chen[2], Fei Tan[1], Jun Wang[1], Jinke Huang[1], Linkun Cai[1], Jiwei Chai[1], Cailing Zhong[1], Yanqiang Shi[4], Wendi Zhang[5,*], Haiyan Zhang[1,3,*], Chongyang Huang[1,3,*]**

[1]Department of Gastroenterology, The Second Affiliated Hospital of Guangzhou University of Chinese Medicine, Guangzhou, China.

[2]Department of Gastroenterology and Hepatology, Guangzhou Key Laboratory of Digestive Diseases, Guangzhou Digestive Disease Center, Guangzhou First People's Hospital, School of Medicine, South China University of Technology, Guangzhou, China.

[3]Guangdong Provincial Key Laboratory of Chinese Medicine for Prevention and Treatment for Refractory Chronic Diseases; State Key Laboratory of Dampness Syndrome of Chinese Medicine; State Key Laboratory of Traditional Chinese Medicine Syndrome, Guangzhou 510120, China.

[4]Institute of Dermatology and Venereology, Dermatology Hospital, Southern Medical University, Guangzhou, China.

[5]Department of Gastroenterology, Guangdong Provincial Key Laboratory of Gastroenterology, Institute of Gastroenterology of Guangdong Province, Nanfang Hospital, Southern Medical University, Guangzhou, China.

†These authors contributed equally to this study.

***Correspondence:** Wendi Zhang (windy101@smu.edu.cn), Ph.D, Department of Gastroenterology, Nanfang Hospital, Southern Medical University, Guangzhou 510515, China. Haiyan Zhang (zhanghaiyan@gzucm.edu.cn), and Chongyang Huang (hcyoung16@gzucm.edu.cn), Ph.D, Department of Gastroenterology, The Second Affiliated Hospital of Guangzhou University of Traditional Chinese Medicine, Guangzhou 510120, China.

**Table S1. LDA scores for the specified Ruminococcus species in cohorts (SRP057027)**

| Phenotypes | Data type | Scientific name | LDA score |
|---|---|---|---|
| Crohn's Disease | mNGS | *Ruminococcus bromii* | -4.407 |
| Crohn's Disease | mNGS | *Ruminococcus sp. 5_1_39BFAA* | -3.559 |
| Crohn's Disease | mNGS | *Ruminococcus lactaris* | -3.311 |
| Crohn's Disease | mNGS | *Ruminococcus callidus* | -3.040 |
| Crohn's Disease | mNGS | *Ruminococcus champanellensis* | -2.753 |

**Table S2. LDA scores for the specified Ruminococcus species in cohorts (PRJEB15371)**

| Phenotypes | Data type | Scientific name | LDA score |
|---|---|---|---|
| Crohn's Disease | mNGS | *Ruminococcus bromii* | -4.575 |
| Crohn's Disease | mNGS | *Ruminococcus sp. 5_1_39BFAA* | -4.053 |
| Crohn's Disease | mNGS | *Ruminococcus lactaris* | -3.782 |
| Crohn's Disease | mNGS | *Ruminococcus callidus* | -3.579 |

**Table S3. LDA scores for the specified Ruminococcus species in cohorts (PRJNA389280)**

| Phenotypes | Data type | Scientific name | LDA score |
|---|---|---|---|
| Crohn's Disease | mNGS | *Ruminococcus bromii* | -3.413 |
| Crohn's Disease | mNGS | *Ruminococcus callidus* | -3.378 |
| Crohn's Disease | mNGS | *Ruminococcaceae bacterium D16* | -3.165 |
| Crohn's Disease | mNGS | *Ruminococcus sp. 5_1_39BFAA* | -2.574 |

**Table S4. LDA scores for the specified Ruminococcus species in cohorts (PRJNA400072)**

| Phenotypes | Data type | Scientific name | LDA score |
|---|---|---|---|
| Crohn's Disease | mNGS | *Ruminococcus bromii* | -4.542 |
| Crohn's Disease | mNGS | *Ruminococcus sp. 5_1_39BFAA* | -4.371 |
| Crohn's Disease | mNGS | *Ruminococcus lactaris* | -3.588 |
| Crohn's Disease | mNGS | *Ruminococcus callidus* | -3.374 |

**Table S5. Clinical Characteristics of CD Patients**

| | Healthy Volunteers | CD patients (active) |
|---|---|---|
| Number | n = 20 | n = 30 |
| Gender (m/f) | 10/10 | 20/10 |
| Age (year) | 25（18-29） | 28 (18-47) |
| CDAI score | - | 220 (175-387) |
| SES-CD | - | 15 (3-45) |
| ESR (mm/h) | - | 42 (3-120) |
| CRP (mg/L) | - | 28.9 (0-257.2) |
| Disease Behavior (Montreal B) | - | B1: 18 (60%)<br>B2: 9 (30%)<br>B3: 3 (10%) |
| Disease Duration (years) | - | 5 (0.5-20) |
| Previous Surgery, n (%) | - | 4 (13.3%) |

**Note:** Values are reported as median and (range). CDAI: Crohn's Disease Activity Index.

**Table S6. Primer sequence of *Hif-1α*$^{fl/fl}$ Vil1-Cre mice**

| | Forward primer | Reverse primer |
|---|---|---|
| PrimerS1 | GGAAAGAATTCTCGGTGTGTATCAT | TACTTCCAGGTGCTTACTTCCTTAG |
| PrimerS2 | GCCTTCTCCTCTAGGCTCGT | AGGCAAATTTTGGTGTACGG |

**Note:** PrimerS1 (for Hif-1a floxed allele): Annealing temperature 60.0°C. Wild type: PCR yields a single 173 bp band; heterozygote: PCR yields 173 bp and 231 bp bands; homozygote: PCR yields a single 231 bp band. PrimerS2 (for *Vil1-Cre* transgene): Annealing temperature 60.0°C. Cre amplicon: PCR yields ~150 bp band (confirms Cre presence).

**Table S7. The detailed information of reagents used in this study.**

| Name | Manufacturer | Product code |
|---|---|---|
| Picrylsulfonic acid solution | Sigma-Aldrich | P2297 |
| Sodium D-lactate | Sigma-Aldrich | 71716 |
| Sodium L-lactate | Sigma-Aldrich | 71718 |
| Oxamic acid sodium | MedChemExpress | HY-W013032A |
| Tenapanor | MedChemExpress | HY-15991 |
| Vancomycin | Sigma-Aldrich | 1404–93-9 |
| Metronidazole | Sigma-Aldrich | 443–48-1 |
| Neomycin sulfate | Sigma-Aldrich | 1405–10-3 |
| Ampicillin | Sigma-Aldrich | 69–52-3 |
| Sodium butyrate | Macklin | 156-54-7 |

**Table S8. Scoring system for inflammation-associated histological changes in TNBS colitis.**

| Score | Histologic changes in TNBS colitis |
|---|---|
| 0 | No evidence of inflammation |
| 1 | Low level of inflammation, with scattered infiltrating mononuclear cells (1–2 foci) |
| 2 | Moderate inflammation, with multiple foci |
| 3 | High level of inflammation, with increased vascular density and marked wall thickening |

| Score | Histologic changes in TNBS colitis |
| --- | --- |
| 4 | Maximal severity of inflammation, with transmural leukocyte infiltration and loss of goblet cells |

**Table S9. Scoring system for inflammation-associated histological changes in Interleukin 10-deficient colitis.**

| | Histological appearance | Score |
| --- | --- | --- |
| **I** | **Enterocyte loss** | |
| | Normal | 0 |
| | Loss of single cell | 1 |
| | Loss of groups of cells | 2 |
| | Frank ulceration | 3 |
| **II** | **Crypt inflammation** | |
| | Normal | 0 |
| | Single inflammatory cell | 1 |
| | Cryptitis | 2 |
| | Crypt abscess | 3 |
| **III** | **Lamina propria mononuclear cells** | |
| | Normal | 0 |
| | Slight increase | 1 |
| | Moderate increase | 2 |
| | Marked increase | 3 |
| **IV** | **Neutrophils** | |
| | Normal | 0 |
| | Slight increase | 1 |
| | Moderate increase | 2 |
| | Marked increase | 3 |
| **V** | **Epithelial hyperplasia** | |
| | Normal | 0 |
| | Mild | 1 |
| | Moderate | 2 |
| | Pseudopolyp | 3 |

**Table S10. The detailed information of antibodies used in this study.**

| Name | Manufacturer | Product code | Host | Dilution | Application |
|---|---|---|---|---|---|
| ZO-1 | Servicebio | GB151981 | Rabbit | 1:400 | IF |
| Occludin | Servicebio | GB111401 | Rabbit | 1:500 | IF |
| MUC2 | Servicebio | GB11344 | Rabbit | 1:500 | IF |
| F4/80 | Servicebio | GB113373 | Rabbit | 1:500 | IF |
| MPO | Servicebio | GB12224 | Rabbit | 1:500 | IF |
| HMGCS2 | Abcam | ab137043 | Rabbit | 1:200, 1:1,000 | IHC,WB |
| HIF-1α | PTMbio | PTM-5851 | Rabbit | 1:150 | IHC |
| Ki67 | Servicebio | GB151499 | Rat | 1:1000 | IF |
| Pcna | Servicebio | GB12010 | Mouse | 1:1000 | IF |
| HCAR2 | Affinity | DF4890 | Rabbit | 1:300 | IHC |
| REG3G | Affinity | DF6869 | Rabbit | 1:300 | IHC |
| CCL20 | proteintech | 84413-3-RR | Rabbit | 1:300 | IHC |
| DUOX2 | Boster | A02186 | Rabbit | 1:300 | IHC |
| sPLA2 | Affinity | DF6366 | Rabbit | 1:300 | IF |
| LGR5 | ABclonal | A10545 | Rabbit | 1:300 | IF |
| FABP1 | ABclonal | A11213 | Rabbit | 1:300 | IF |
| CHGA | ABclonal | A9576 | Rabbit | 1:300 | IF |
| Ki67 | Servicebio | GB151499 | Rat | 1:1000 | IF |
| Goat anti-rabbit secondary antibody | Servicebio | G1213 | Goat | 1:200 | IHC |
| anti-β-actin | Proteintech | 81115-1-RR | Rabbit | 1:10,000 | WB |
| anti-GAPDH | Cell Signaling Technology | 5174T | Rabbit | 1:1,000 | WB |
| goat anti-rabbit IgG-HRP | Proteintech | SA00001-1 | Goat | 1:10,000 | WB |
| α-SMA | Abmart | T55295 | Rabbit | 1:2,000 | WB |
| fibronectin | Abmart | T59537 | Rabbit | 1:1,000 | WB |
| HIF-1α | Abcam | ab216842 | Rabbit | 1:1,000 | WB |
| PPARα | Abcam | ab314112 | Rabbit | 1:1,000 | WB |
| Anti-L-Lactyl Lysine | PTMbio | PTM-1401RM | Rabbit | 1:1,000 | WB |
| Anti-Lactyl-H3 (Lys9) | PTMbio | PTM-1419RM | Rabbit | 1:1,000 | WB |
| Anti-Lactyl-H3 (Lys14) | PTMbio | PTM-1414RM | Rabbit | 1:1,000 | WB |
| Anti-Lactyl-H3 (Lys18) | PTMbio | PTM-1427RM | Rabbit | 1:1,000 | WB |
| Anti-Lactyl-H4 (Lys5) | PTMbio | PTM-1407RM | Rabbit | 1:1,000 | WB |
| Anti-Lactyl-H4 | PTMbio | PTM- | Rabbit | 1:1,000 | WB |

| | | | | | |
|---|---|---|---|---|---|
| (Lys8) | | 1415RM | | | |
| Anti-Lactyl-H4 (Lys12) | PTMbio | PTM-1411RM | Rabbit | 1:1,000 | WB |
| Anti-Lactyl-H4 (Lys16) | PTMbio | PTM-1417RM | Rabbit | 1:1,000 | WB |
| NHE-3(SLC9A3) | Wanleibio | WL 00814 | Rabbit | 1:1,000 | WB |

**Abbreviations:** WB: western blot; IHC: immunohistochemistry; IF: immunofluorescence.

**Table S11. Mouse primer sequences for RT-qPCR.**

| Gene | Forward primer (5'→3') | Reverse primer (5'→3') |
|---|---|---|
| *Rn18s* | CGATCCGAGGGCCTCACTA | AGTCCCTGCCCTTTGTACACA |
| *HK2* | CCCTGTGAAGATGTTGCCCACT | CCTTCGCTTGCCATTACGCACG |
| *Eno1* | TGCGTCCACTGGCATCTAC | CAGAGCAGGCGCAATAGTTTTA |
| *Pkm* | GTGGCTCGGCTGAATTTCTCT | CACCGCAACAGGACGGTAG |
| *Ldha* | TGTCTCCAGCAAAGACTACTGT | GACTGTACTTGACAATGTTGGGA |
| *Pfkfb3* | TCATCGAGTCGGTCTGTGACGA | CATGGCTTCTGCTGAGTTGCAG |
| *Duoxa2* | GACGGGGTGCTACCCTTTTAC | GCTAAGAAGGACTCTCACCAAC |
| *Duox2* | AACCACCTATCTGGGCATCATCCT | AGCTGCCATGGATGATGATCTGGA |
| *Reg3g* | ACATCAACTGGGAGACGAATC | TTTGGGATCTTGCTTGTGGCTA |
| *Pfk* | AGGAGGGCAAAGGAGTGTTT | TTGGCAGAAATCTTGGTTCC |
| *Ccl5* | CTGCTGCTTTGCCTACCTCT | CGAGTGACAAACACGACTGC |
| *Ccl2* | CCTGCTGTTCACAGTTGCC | ATTGGGATCATCTTGCTGGT |
| *Cxcl10* | CCAAGTGCTGCCGTCATTTTC | GGCTCGCAGGGATGATTTCAA |
| *Cxcl1* | ACCCAAACCGAAGTCATAGC | TCTCCGTTACTTGGGGACAC |
| *Tnf* | CCACCACGCTCTTCTGTCTAC | AGGGTCTGGGCCATAGAACT |
| *Il1b* | TGCCACCTTTTGACAGTGATG | TGATGTGCTGCTGCGAGATT |
| *Il6* | TAGTCCTTCCTACCCCAATTTCC | TTGGTCCTTAGCCACTCCTTC |
| *Nos2* | CGGCAAACATGACTTCAGGC | GCACATCAAAGCGGCCATAG |
| *Ccl4* | CATGAAGCTCTGCGTGTCTG | GAAACAGCAGGAAGTGGGAG |
| *Ccl20* | CGACTGTTGCCTCTCGTACA | GAGGAGGTTCACAGCCCTTT |
| *Timp1* | CGAGACCACCTTATACCAGCG | ATGACTGGGGTGTAGGCGTA |
| *Mmp9* | CTTCGACACTGACAAGAAGTGG | GGCACGCTGGAATGATCTAAG |
| *Acta2* | GGTACCACCATGTACCCAGG | CACAGTTGTGTGCTAGAGGC |
| *Tgfb1* | CTCCCGTGGCTTCTAGTGC | GCCTTAGTTTGGACAGGATCTG |
| *Ctgf* | AGACCTGTGCCTGCCATTAC | ACGCCATGTCTCCGTACATC |
| *Fn1* | ATGTGGACCCCTCCTGATAGT | GCCCAGTGATTTCAGCAAAGG |
| *Col1a1* | TGTTCAGCTTTGTGGACCTC | GGTTTCCACGTCTCACCATT |
| *Col3a1* | CTGTAACATGGAAACTGGGGAAA | CCATAGCTGAACTGAAAACCACC |
| *Cpt1* | TCGAAACATCTACCATGCAGCA | CAGCATTCTTCGTGACGTTGG |
| *Cpt2* | TGACCGACACTTGTTTGCTC | TGGTTTATCCGCTGGTATGC |
| *Mcad* | GATCGCAATGGGTGCTTTTGATAGAA | AGCTGATTGGCAATGTCTCCAGCAAA |
| *Acox1* | AGATAATTGGCACCTACGCC | CATGTAACCCGTAGCACTCC |

| | | |
|---|---|---|
| *Fgf21* | CTGGGGGTCTACCAAGCATA | CACCCAGGATTTGAATGACC |
| *Fgfr1* | GATGACCTCACCGCTCTACC | GGAAGTCGCTCTTCTTGGTG |
| *Ehhadh* | TTGGACCATACGGTTAGAGC | ATGTAAGGCCAGTGGGAGAT |
| *Slc27a2* | GGAGTCGTGGAGGTCTGAAG | GCGATGATGATTGATGGTTG |
| *Hmgcs2* | ATACCACCAACGCCTGTTATGG | CAATGTCACCACAGACCACCAG |
| *Ppara* | CGAGGTGAAAGATTCGGAAA | GGCCTTGACCTTGTTCATGT |

**Table S12. Bacterial primers for qPCR**

| Target | Forward Primer (5'→3') | Reverse Primer (5'→3') |
|---|---|---|
| *Total Bacteria* | TCCTACGGGAGGCAGCAGT | GACTACCAGGGTATCTAATCCTGTT |
| *Lactobacillus* | AGCAGTAGGGAATCTTCCA | CACCGCTACACATGGAG |
| *Clostridium* | CGTCAGATCATCATGCCCCTTACG | GTATGTCRCAAGCGTTATCC |
| *Ruminococcus albus* | GTTTTAGGATTGTAAACCTCTGTCTT | CCTAATATCTACGCATTTCACCGC |
| *Ruminococcus callidus* | CTACACACGTACTACAATGGCAATATAACA | TTACCTCACCGACTTCGGGTGT |
| *Ruminococcus sp. 5_1_39BFAA* | CCTACGGGAGGCAGCAG | GGACTACAGGGTATCTAAT |
| *[Ruminococcus] lactaris* | ACGGCAGAGTAATGTCTGCTT | CATCTCACGACACGAGCTGA |
| *Ruminococcus bromii* | GGGGTTTGCTCCACATCACT | ACAAAACGGAGGAAGGTGGG |
| *Butyricicoccus pullicaecorum* | ACCTGAAGAATAAGCTCC | GATAACGCTTGCTCCCTACGT |
