# Supplementary material for: Gut Lactate Boosts Ruminococcus via Histone Lactylation to Mediate Time‐Restricted Feeding Protection in Crohn's Disease
Source: Adv Sci (Weinh). 2026 Apr 7;13(33):e18419. doi: 10.1002/advs.202518419 (PMC13271634; doi:10.1002/advs.202518419)
Supplement: Supplementary file 3 — Supporting File 3: advs75028‐sup‐0003‐Method.pdf. [file ADVS-13-e18419-s001.pdf]

Methods

Single-cell RNA sequencing of mouse colon tissue

Single-cell suspensions were prepared from mouse colon tissue by mechanical dissociation followed by enzymatic digestion. Cell viability was assessed using trypan blue exclusion and exceeded 90%. The suspensions were filtered through a 30 μm nylon mesh, and live cell concentration was adjusted to 1,000–2,000 cells/μL using a Countess® II Automated Cell Counter. Single-cell capture, barcoding, and library construction were performed using the DNBelab C series microfluidic system (MGI), which co-encapsulates cells with barcoded beads and index carrier beads for droplet-based mRNA capture and reverse transcription, followed by cDNA amplification. The resulting libraries were sequenced on a DNBSEQ-T7RS platform (MGI) with PE150 strategy. Bioinformatics processing and downstream analysis were supported by the online platform OMICSMART (https://www.omicsmart.com) provided by GENE DENOVO (Guangzhou, China).

Fecal lactate measurement

Fecal samples from female and refed mice were collected, snap-frozen, and stored at −80°C. D-lactate concentrations were quantified using the D-Lactate Assay Kit (Beyotime, S0204) according to the manufacturer's instructions. Samples were homogenized in Beyolysis™ Buffer A, centrifuged, and the supernatant was assayed in a 96-well plate. The assay is based on D-lactate dehydrogenase-coupled reduction of WST 8 to formazan, with absorbance read at 450 nm. A standard curve (0-1 mM D-lactate) was used for calculation, and results were normalized to fecal weight.

The chronic colitis model

The chronic TNBS-induced colitis model was established following a published protocol[1]. Briefly, the TNBS sensitization solution was prepared by mixing acetone/olive oil (4:1, v/v) with 5% (w/v) TNBS at a 4:1 ratio (v/v), yielding 2.5% TNBS. For colitis induction, the working solution was prepared by combining equal volumes of 5% (w/v) TNBS and anhydrous ethanol. Male C57BL/6J mice (6-8 weeks old) were epidermally sensitized with 100 μL sensitization solution on shaved dorsum. Seven days post-sensitization, chronic inflammation

was induced via intrarectal administration of 80 μL TNBS-ethanol solution under isoflurane anesthesia, followed by weekly repeated administrations for six consecutive weeks. Body weight and survival were monitored daily. Prior to colitis induction, mice underwent a 4-week time-restricted feeding (TRF) regimen with the following groups: ND TNBS, TRF-12h TNBS, TRF-16h TNBS and TRF-20h TNBS; TRF was maintained throughout the 6-week modeling period. At the end of the intervention period, mouse colonic tissues were collected for transcriptomic, histopathological, and molecular biology analyses. To investigate whether the effects of TRF intervention are influenced by sex, the same model was established in female C57BL/6J mice (6–8 weeks old). The modeling procedure was identical to that used in males, with the ND TNBS and TRF 12h TNBS groups established.

To investigate whether lactate mediates the anti-inflammatory effects of TRF by enriching *Ruminococcus* and mitigating systemic inflammation, wild-type male C57BL/6J mice (6–8 weeks old) were randomly assigned to six groups: ND: normal diet, *ad libitum* feeding; TRF-12h: time-restricted feeding (12-h feeding/12-h fasting); TRF-12h + oxamate: TRF-12h regimen + daily intraperitoneal injection of oxamate (750 mg/kg), a lactate dehydrogenase inhibitor; ND TNBS: normal diet + TNBS-induced colitis; TRF-12h + TNBS: TRF-12h regimen + TNBS-induced colitis; TRF-12h + oxamate + TNBS: TRF-12h + daily oxamate injection + TNBS-induced colitis. Body weight, fecal samples and colon tissues were collected for analysis of lactate levels, gut microbiota (*Ruminococcus* abundance), inflammatory markers, and histopathology.

To investigate the role of SLC9A3 in TRF-mediated Ruminococcus enrichment and barrier protection, wild-type male C57BL/6J mice (6–8 weeks old) were randomly assigned to seven groups: (1) ND: normal diet, *ad libitum* feeding; (2) TRF-12h: time-restricted feeding (12-h feeding/12-h fasting); (3) TRF-12h + Tenapanor: TRF-12h regimen + daily oral gavage of Tenapanor (0.25 mg/kg), the SLC9A3 inhibitor[2]; (4) ND + TNBS: normal diet + TNBS-induced colitis; (5) TRF-12h + TNBS: TRF-12h regimen + TNBS-induced colitis; (6) TRF-12h + Tenapanor + TNBS: TRF-12h regimen + daily Tenapanor gavage + TNBS-induced colitis. (7) TRF-12h + Tenapanor + TNBS+ *R.bromii*: TRF-12h regimen + daily Tenapanor gavage + TNBS-induced colitis+ daily *R.bromii* gavage. Body weight and colon tissues were collected for analysis of inflammatory markers and

histopathology.

Fecal microbiota transplantation (FMT) was performed to directly test the causal role of the TRF-conditioned gut microbiota in conferring protection against colitis. Fresh fecal samples were collected from donor mice in the ND and TRF_12h groups. The samples were processed under sterile conditions by resuspending feces in PBS at a concentration of 0.125 g/mL. Prior to FMT, recipient mice were pretreated with a broad-spectrum antibiotic cocktail to deplete their endogenous gut microbiota. The antibiotic regimen, administered intragastrically once daily for 5 days, consisted of ampicillin (200 mg/kg), neomycin sulfate (200 mg/kg), metronidazole (200 mg/kg), and vancomycin (100 mg/kg). Following depletion, recipient mice received 0.15 mL of the prepared donor fecal suspension via oral gavage once daily for 5 days. Subsequently, the chronic TNBS colitis model was induced as described previously. During the colitis induction period, mice received a maintenance administration of the corresponding fecal suspension via oral gavage every three days until the end of the experiment.

TNBS-induced acute colitis models

To investigate the role of lactate during "fasting-refeeding" in modulating the gut environment and TNBS-induced colitis, 6–8-week-old male C57BL/6J mice were divided into four groups: Control, Refeed (12-h fast/12-h refeed), Refeed+ABX, and Refeed+ABX+lactate (300 mg/kg, i.g.). Four hours after refeeding began, groups 3 and 4 received an oral antibiotic cocktail (with group 4 also receiving lactate) to deplete gut microbiota. Eight hours post-treatment, fecal samples were collected from all mice. After fecal collection, acute colitis was induced via intrarectal TNBS administration. At the designated endpoint, colon tissues were collected for subsequent analysis.

To determine whether the protective effect of butyrate against colitis requires epithelial HIF-1α, littermate C57BL/6J mice (6–8 weeks old) were randomly assigned to four experimental groups: (1) HIF-1$\alpha^{Fl/Fl}$ + TNBS, (2) HIF-1$\alpha^{\Delta IEC}$ + TNBS, (3) HIF-1$\alpha^{Fl/Fl}$ + TNBS + butyrate (sodium butyrate, 300 mg/kg), and (4) HIF-1$\alpha^{\Delta IEC}$ + TNBS + butyrate. Acute colitis was induced with TNBS as described previously. Colon tissues were harvested at the experimental endpoint for analysis.

RNA Extraction and Quantitative RT-qPCR

The colon tissue processing was performed as follows: (1) Following the chronic TNBS regimen, the entire colon was excised, gently flushed with cold PBS, and opened longitudinally. (2) The primary fibrotic stricture in the distal colon was then identified based on definitive macroscopic features (palpable thickening, rigidity, and/or stenosis). (3) Finally, from the core of this contiguous fibrotic area, three adjacent full-thickness tissue strips were collected, pooled together, immediately flash-frozen as a single combined sample in liquid nitrogen, and stored at –80°C until processing.

Quantitative reverse transcription PCR (RT-qPCR) was performed to evaluate transcriptional changes in inflammatory mediators, fibrotic markers, and metabolic regulators. Total RNA was extracted from snap-frozen colon tissues and cells using TRIzol® Reagent (AGBIO, Hunan, China; AG21101), followed by cDNA synthesis with RT-gDNA Digestion Mix (Yeasen, Shanghai, China; 11151ES60). PCR amplification was performed using Hieff UNICON® qPCR SYBR Green Master Mix (Yeasen, Cat# 11185ES08) on a LightCycler® 96 System(Roche). Gene expression levels were calculated using the $2^{\wedge-\Delta\Delta Ct}$ method. 18S rRNA was used as the reference gene for normalization.

Reference

1 Wirtz, S., Popp, V., Kindermann, M. *et al.* Chemically induced mouse models of acute and chronic intestinal inflammation. *Nature Protocols* (2017) **12**: 1295-1309, doi:10.1038/nprot.2017.044.

2 King, A. J., Siegel, M., He, Y. *et al.* Inhibition of sodium/hydrogen exchanger 3 in the gastrointestinal tract by tenapanor reduces paracellular phosphate permeability. *Science Translational Medicine* (2018) **10**, doi:10.1126/scitranslmed.aam6474.
